# Diagnostic performance of circulating microRNAs for the early diagnosis of different types of acute cerebrovascular disease

**DOI:** 10.1371/journal.pone.0359648

**Published:** 2026-10-05

**Authors:** Sharbanu Battakova, Marina Grigolashvili, Irina Kadyrova, Dmitry Kasatkin, Mira Beisembayeva, Shynar Muratbekova, Yelena Shayakhmetova, Altyn Bakenova, Alina Koshelyuk

**Affiliations:** 1 Department of Neurology, Psychiatry and Rehabilitation, Karaganda Medical University, Karaganda, Kazakhstan; 2 Institute of Life Sciences, Karaganda Medical University, Karaganda, Kazakhstan; 3 Department of Neurology, Yaroslavl State Medical University, Yaroslavl, Russia; 4 Scientific Research Laboratory, Karaganda Medical University, Karaganda, Kazakhstan; University of Catania, ITALY

## Abstract

Early differentiation of acute cerebrovascular conditions is important for timely clinical management, while blood-based markers that could complement established diagnostic approaches remain under investigation. This exploratory case-control study evaluated the circulating expression and diagnostic performance of seven candidate microRNAs (miRNAs) in acute ischemic stroke (AIS), hemorrhagic stroke (HS), and transient ischemic attack (TIA) during the first 12 h after symptom onset. The study included 200 participants: 50 patients with AIS, 50 with HS, 50 with TIA, and 50 controls. Plasma was collected from the clinical groups at 3–4.5 h and 12 h after symptom onset and from controls at a single time point. Expression levels of let-7e-5p, miR-21-5p, miR-107, miR-125b-5p, miR-130a-3p, miR-143-3p, and miR-222-3p were measured using real-time quantitative polymerase chain reaction (RT-qPCR). Diagnostic performance was evaluated using receiver operating characteristic (ROC) curve analysis and binary logistic regression. let-7e-5p showed the most consistent expression pattern, with lower levels in AIS, HS, and TIA than in controls at both sampling time points. miR-107 was lower in AIS and HS than in controls at both time points, whereas miR-125b-5p showed a predominantly AIS-related pattern. Among comparisons with controls, let-7e-5p yielded the highest individual area under the ROC curve (AUC), reaching 0.867 for AIS at 12 h, whereas miR-222-3p yielded the highest individual AUC for AIS versus HS (0.833 at 12 h). Multi-miRNA models did not significantly improve discrimination over the best-performing individual markers. In the complete-case subset, adding let-7e-5p to a model comprising routinely available clinical variables was associated with a higher AUC for AIS versus controls (0.982) than either component alone. Overall, the observed expression and discrimination patterns support further investigation of circulating miRNAs as complementary markers in acute cerebrovascular disease. Prospective evaluation in larger, independent cohorts, including patients with stroke-mimicking conditions, is needed to determine their diagnostic performance and incremental value in real-world clinical settings.

## Introduction

Acute cerebrovascular disease remains a leading cause of mortality and long-term disability worldwide [1]. Despite substantial advances in neuroimaging and reperfusion therapies, timely assessment of patients with suspected stroke remains clinically challenging. The effectiveness of reperfusion strategies, including intravenous thrombolysis and mechanical thrombectomy, is strongly time-dependent, emphasizing the importance of rapid and accurate evaluation after symptom onset [2]. Differentiation among AIS, HS, and TIA is particularly important during the early phase, when clinical manifestations may overlap. Molecular biomarkers that provide information complementary to established diagnostic approaches may therefore have potential value in the evaluation of acute cerebrovascular disease [3].

Clinical assessment and neuroimaging remain central to the diagnosis and classification of AIS, HS, and TIA. Non-contrast computed tomography (CT) is the first-line imaging modality for excluding intracranial hemorrhage, but its sensitivity for early ischemic changes may be limited during the first hours after symptom onset [4]. In one study, magnetic resonance imaging (MRI) demonstrated a diagnostic accuracy of 89% relative to the final clinical diagnosis of stroke, compared with 54% for CT [5]. However, the use of MRI in emergency settings may be constrained by availability, acquisition time, and contraindications in some patients [6]. These considerations have stimulated continued investigation of blood-based biomarkers that could complement clinical and imaging findings, particularly during the early assessment of patients with suspected cerebrovascular events.

A broad range of laboratory biomarkers has been investigated in stroke, but their diagnostic performance and clinical applicability remain under evaluation [7]. Among these, circulating miRNAs have attracted increasing interest. miRNAs are short non-coding ribonucleic acid (RNA) molecules that regulate gene expression at the post-transcriptional level [8]. Their relative stability in plasma, together with reported alterations in circulating levels following cerebrovascular events, has prompted investigation of their potential as blood-based markers of stroke [9]. However, substantial variability across studies has been associated with differences in study populations, sampling windows, biological matrices, normalization strategies, and analytical methods, complicating the interpretation and comparison of reported findings.

The seven candidate miRNAs investigated in the present study were selected a priori based on previous evidence of altered circulating expression in cerebrovascular disease and their reported involvement in pathways relevant to cerebral and vascular injury. let-7e-5p has been associated with the regulation of endothelial progenitor cell function, angiogenesis, and vascular repair [10], whereas miR-21-5p has been implicated in interleukin-6/interleukin-6 receptor (IL-6/IL-6R) signaling and ischemic endothelial cell injury [11]. miR-107 has been linked to phosphoinositide 3-kinase/protein kinase B (PI3K/AKT)-dependent pathways involved in neuronal survival and apoptosis [12], while miR-125b-5p has been proposed to regulate the fibroblast growth factor 2/mitogen-activated protein kinase (FGF2/MAPK) pathway during cerebral ischemic injury [13]. miR-130a-3p has been associated with neuronal morphology and amyloid precursor protein (APP) expression [14], and miR-143 with neuronal energy metabolism through targeting hexokinase 2 (HK2) [15]. miR-222 has also been implicated in the regulation of inflammation and apoptosis following HS through negative regulation of integrin beta-8 (ITGB8) [16]. Despite these experimental and clinical observations, comparative evidence on the circulating expression and discriminatory performance of these miRNAs across AIS, HS, and TIA during the first hours after symptom onset remains limited.

The present study therefore aimed to evaluate the circulating expression and diagnostic performance of seven candidate miRNAs (let-7e-5p, miR-21-5p, miR-107, miR-125b-5p, miR-130a-3p, miR-143-3p, and miR-222-3p) in patients with AIS, HS, and TIA at 3–4.5 h and 12 h after symptom onset. We further examined differences in miRNA expression across the clinical groups, within-patient changes between the two sampling time points, and associations with clinical, demographic, and laboratory characteristics. In addition, we explored the discriminatory performance of individual and combined miRNAs and assessed whether incorporating miRNA measurements into a model based on routinely available clinical variables provided additional discriminatory information.

## Materials and methods

### Study design and participants

Participants were recruited from two stroke centers in Karaganda, Kazakhstan, between January 2024 and June 2025. Patients with acute cerebrovascular disease admitted within the first hours after symptom onset were eligible for inclusion. A total of 200 participants were enrolled into four groups of 50 participants each: AIS, HS, TIA, and controls. Sample size was calculated a priori using G*Power version 3.1.9.7. The minimum required sample size was 32 participants per group (effect size = 0.30, α = 0.05, power = 80%). A larger sample was recruited to account for potential attrition and increase the robustness of the analyses. Diagnoses of AIS, HS, and TIA were established based on clinical presentation, neurological examination, and neuroimaging findings, including brain CT and/or MRI, in accordance with current international and national clinical guidelines [17–20].

Inclusion criteria were age ≥ 18 years, a confirmed diagnosis of AIS, HS, or TIA, hospital admission within 4.5 h of symptom onset, a National Institutes of Health Stroke Scale (NIHSS) score of 3–23 at admission [21], and written informed consent. Exclusion criteria included active malignancy, acute infectious or inflammatory disease, severe systemic disorders, and refusal to participate. Demographic, clinical, and laboratory data, including vascular risk factors and comorbidities, were collected for all participants. Neurological deficit was assessed at admission using the NIHSS, and lesion location was determined by CT and/or MRI. The etiological subtype of AIS was classified according to the Trial of Org 10172 in Acute Stroke Treatment (TOAST) criteria [22].

For patients with TIA, the NIHSS score reported in the study was obtained at hospital admission, when focal neurological symptoms were still present. TIA was defined as a transient focal neurological deficit that resolved completely within 24 h, with no evidence of acute cerebral infarction on CT and/or MRI. All patients ultimately classified as having TIA had complete resolution of neurological deficits and an NIHSS score of 0 at 24 h. The final diagnosis was established by a neurologist based on the clinical course and neuroimaging findings.

The control group comprised ambulatory patients recruited from the City Center for Primary Health Care and matched to the patient groups by age, sex, and hypertension status. Eligibility criteria for controls included age ≥ 18 years, absence of acute neurological symptoms or focal neurological deficits, no history of stroke or TIA, and written informed consent. Neuroimaging was not routinely performed in controls in the absence of clinical signs suggestive of acute cerebrovascular disease. The same exclusion criteria were applied to controls as to the patient groups.

To minimize potential sources of bias, consecutive eligible patients were enrolled according to predefined eligibility criteria, and all blood samples were processed using the same standardized laboratory protocol. The study was conducted and reported in accordance with the Strengthening the Reporting of Observational Studies in Epidemiology (STROBE) guidelines.

### Plasma sample collection and processing

Peripheral venous blood samples were collected from patients with acute cerebrovascular disease at two time points: 3–4.5 h and 12 h after symptom onset. The second sample was collected regardless of whether reperfusion therapy had been performed. A single blood sample was obtained from each control participant and used as the reference for comparisons at both patient sampling time points. For each collection, 10 mL of peripheral venous blood was drawn into vacuum tubes containing K2-ethylenediaminetetraacetic acid (K2-EDTA) as the anticoagulant. Within 15 min of venipuncture, samples were centrifuged at room temperature for 15 min at 1,600 × g (3,000 rpm) to separate plasma from cellular components, following protocols reported in previous studies of circulating miRNAs [23–25].

Hemolysis was assessed by measuring the concentration of cell-free hemoglobin in plasma [26]. Hemolyzed samples were excluded, and recruitment continued until the predefined sample size was reached. Plasma was transferred into sterile plastic microcentrifuge tubes, aliquoted, and stored at −80 °C until analysis. Repeated freeze–thaw cycles were avoided. All samples were assigned coded identifiers before laboratory analysis, and personnel performing RNA extraction and RT-qPCR were blinded to participants’ clinical diagnoses and group assignments.

### RNA extraction from plasma

Total RNA was extracted from 100 μL of plasma using the MagMAX™ mirVana™ Total RNA Isolation Kit (Thermo Fisher Scientific, Waltham, MA, USA) according to the manufacturer’s instructions. RNA concentration and purity were assessed spectrophotometrically using a NanoPhotometer® P330 (Implen, Munich, Germany), with purity evaluated based on the A260/A280 and A260/A230 absorbance ratios. RNA integrity was assessed by microfluidic capillary electrophoresis using the LabChip GX II Touch system (PerkinElmer, Waltham, MA, USA). Extracted RNA was either used immediately for reverse transcription or stored at −80 °C until use.

### Reverse transcription (RT) and RT-qPCR

The seven candidate miRNAs were selected a priori based on the findings of a previously published systematic review [27] and the available literature, focusing on circulating miRNAs with previously reported differential expression and potential diagnostic relevance in acute cerebrovascular disease. Complementary DNA (cDNA) was synthesized from 2 μL of extracted RNA using the TaqMan™ Advanced miRNA cDNA Synthesis Kit (Thermo Fisher Scientific, Waltham, MA, USA) according to the manufacturer’s instructions. Before RT, synthetic cel-miR-39 was added to each RNA sample as an exogenous control to monitor the efficiency of RT and subsequent amplification. The final reaction volume after the miRNA amplification (miR-Amp) step was 50 μL.

miRNA expression was quantified by RT-qPCR using TaqMan™ Fast Advanced Master Mix and individual TaqMan™ Advanced miRNA Assays (Thermo Fisher Scientific, Waltham, MA, USA); assay identification numbers are provided in S1 Table. Amplification was performed on a QuantStudio™ 5 Real-Time PCR System (Thermo Fisher Scientific, Waltham, MA, USA) in a total reaction volume of 20 μL. Thermal cycling consisted of enzyme activation at 95 °C for 20 s, followed by 40 cycles of denaturation at 95 °C for 1 s and annealing/extension at 60 °C for 20 s. Cycle threshold (Ct) values were determined using QuantStudio™ Design and Analysis Software (Thermo Fisher Scientific, Waltham, MA, USA) with automatic baseline and threshold settings. A no-template control (NTC) was included in each amplification run to monitor potential contamination, while a no-reverse-transcription (no-RT) control was used to detect signals arising independently of RT. Neither negative control showed detectable amplification.

miRNA expression was normalized to the endogenous reference miR-16-5p, which has previously been used for normalization of circulating miRNAs in stroke studies [28,29]. Ct values for miR-16-5p across the study groups are presented in S2 Table. For each sample, ΔCt was calculated as Ct(target miRNA) − Ct(miR-16-5p). For relative expression analysis, ΔΔCt was calculated by subtracting the median ΔCt of the control group from the individual ΔCt value, and fold change (FC) was determined using the 2^ − ΔΔCt method. cel-miR-39 was not used for normalization. All inferential analyses of miRNA expression were based on individual ΔCt values. The distribution of ΔCt values was assessed using the Shapiro–Wilk test, and no observations were excluded solely on the basis of extreme ΔCt values.

### Statistical analysis

Statistical analyses were performed using IBM SPSS Statistics for Windows, Version 32.0 (IBM Corp., Armonk, NY, USA). ROC curve analyses, combined logistic regression models, and data visualization were performed in R version 4.6.0 (R Foundation for Statistical Computing, Vienna, Austria). Missing data were not imputed; analyses followed an available-case approach, with observations containing missing values excluded only from analyses involving the corresponding variables. Continuous variables are presented as median and interquartile range (IQR), and categorical variables as frequencies and percentages.

Comparisons between two independent groups were performed using the Mann–Whitney U test, and comparisons among three or more independent groups using the Kruskal–Wallis test. When the latter indicated significant differences, post hoc pairwise comparisons were conducted using Dunn’s test with Holm–Bonferroni correction. For pairwise comparisons of miRNA expression, P values were adjusted across the six group comparisons within each miRNA and sampling time point using the Holm–Bonferroni procedure. As a sensitivity analysis addressing the broader multiple-testing burden, the Benjamini–Hochberg false discovery rate (FDR) procedure was additionally applied across all 84 pairwise tests. Within-group changes in miRNA expression between 3–4.5 h and 12 h were assessed using the Wilcoxon signed-rank test; control measurements were used only for cross-sectional comparisons at each patient sampling time point. Categorical variables were compared using Pearson’s χ² test or Fisher’s exact test when expected cell counts were <5.

Diagnostic models were constructed using binary logistic regression. Before model fitting, multicollinearity among independent variables was assessed using the variance inflation factor (VIF) and tolerance; no substantial multicollinearity was detected (all VIFs < 5). Covariates for the clinical model were selected a priori based on their established clinical relevance to cerebrovascular and cardiovascular disease and their potential association with circulating miRNA profiles, rather than solely on statistical significance in univariable comparisons. The clinical model included age, sex, atrial fibrillation, blood glucose, and total cholesterol. The incremental diagnostic value of let-7e-5p was evaluated by adding it to the clinical model. Model discrimination was assessed using ROC curve analysis, with AUCs and 95% confidence intervals (CIs) calculated. Optimal cutoffs were determined using the Youden index, with corresponding sensitivity and specificity reported. AUCs were compared using DeLong’s test. Improvement in model fit following the addition of let-7e-5p was evaluated using a likelihood ratio test comparing the nested clinical and combined models. Comparisons among the clinical, let-7e-5p, and combined models were restricted to a common complete-case subset with data available for all model variables.

Internal validation using 1,000 bootstrap resamples was performed for the clinical and combined models discriminating AIS from controls and for the miR-222-3p model discriminating AIS from HS at 12 h. Optimism was estimated as the mean difference between the AUC obtained in each bootstrap sample and the AUC obtained when the corresponding bootstrap-fitted model was applied to the original dataset. This estimate was subtracted from the apparent AUC to obtain the optimism-corrected AUC.

Spearman’s rank correlation was used to assess associations between miRNA expression and modified Rankin Scale (mRS) scores at discharge, as well as clinical, demographic, and laboratory characteristics. Functional outcomes were additionally examined by comparing miRNA expression between patients with favorable (mRS 0–2) and unfavorable (mRS 3–6) outcomes using the Mann–Whitney U test, separately for the AIS and HS groups. Correlation analyses were stratified by clinical group and sampling time point. P values from these exploratory analyses were adjusted for multiple testing using the Benjamini–Hochberg FDR procedure. All statistical tests were two-sided, with P < 0.05 considered statistically significant.

### Ethics statement

The study was approved by the Local Bioethics Commission of Karaganda Medical University (Protocol No. 11, October 10, 2023). All procedures involving human participants were conducted in accordance with the ethical principles of the Declaration of Helsinki. Written informed consent was obtained from all participants or their legally authorized representatives prior to enrollment in the study.

## Results

Three participants were excluded from the final analysis because hemolysis was detected in their first plasma samples. Repeat blood sampling was not performed because the predefined 3–4.5-h sampling window had elapsed. The final study population comprised 200 participants, with 50 in each study group.

### Clinical, demographic, and laboratory characteristics of the study groups

The characteristics of the study population are presented in Table 1. Median age ranged from 63.5 to 67.5 years and did not differ significantly among the four groups (P = 0.767). Sex distribution was also similar across groups, with women accounting for 34.0% of participants in the control, AIS, and HS groups and 36.0% in the TIA group. Onset-to-sampling times did not differ significantly among the three clinical groups. The median time to the first blood sampling was 3.8 h (IQR, 3.4–4.2) in the AIS group, 3.7 h (IQR, 3.3–4.1) in the HS group, and 3.9 h (IQR, 3.5–4.2) in the TIA group (P = 0.126). At the second sampling time point, the corresponding values were 12.0 h (IQR, 11.9–12.2), 12.0 h (IQR, 11.9–12.1), and 12.0 h (IQR, 11.9–12.2), respectively (P = 0.695).

**Table 1:** Clinical, demographic, and laboratory characteristics of the study population.

Variable	Control (n = 50)	AIS (n = 50)	HS (n = 50)	TIA (n = 50)	P value
**Demographic characteristics**					
Age, years	64.5 (56.0–74.0)	65.5 (55.0–75.0)	63.5 (53.25–71.75)	67.5 (54.0–74.0)	0.767
Female, n (%)	17 (34.0%)	17 (34.0%)	17 (34.0%)	18 (36.0%)	0.996
**Clinical characteristics**					
NIHSS score at admission	—	8.5 (4.0–13.0)	10.0 (7.25–14.75)	3 (3.0–6.0)	**<0.001**
Hypertension, n (%)	50 (100.0%)	49 (98.0%)	50 (100.0%)	50 (100.0%)	0.389
Diabetes mellitus, n (%)	7 (14.0%)	9 (18.0%)	10 (20.0%)	12 (24.0%)	0.639
Atrial fibrillation, n (%)	3 (6.0%)	13 (26.0%)	4 (8.0%)	4 (8.0%)	**0.006**
Coronary artery disease, n (%)	7 (14.0%)	14 (28.0%)	14 (28.0%)	20 (40.0%)	**0.037**
Previous myocardial infarction, n (%)	6 (12.0%)	8 (16.0%)	2 (4.0%)	3 (6.0%)	0.152
Chronic heart failure, n (%)	20 (40.0%)	18 (36.0%)	24 (48.0%)	12 (24.0%)	0.092
Obesity, n (%)	10 (20.0%)	18 (36.0%)	16 (32.0%)	9 (18.0%)	0.110
Previous stroke, n (%)*	0 (0.0%)	8 (16.0%)	6 (12.0%)	3 (6.0%)	**0.024**
**Laboratory parameters**					
Hemoglobin, g/L	141.00 (133.10–154.95)	135.05 (124.25–152.25)	140.00 (124.25–152.25)	147.70 (133.68–160.25)	0.268
Leukocyte count, × 10^9^/L	6.40 (5.47–7.60)	7.40 (6.70–8.88)	8.07 (6.81–11.74)	8.27 (6.63–10.50)	**<0.001**
Platelet count, × 10^9^/L	222.40 (194.98–288.00)	215.80 (187.75–249.53)	223.60 (193.35–302.30)	245.00 (213.00–275.80)	0.074
Blood glucose, mmol/L	5.65 (4.90–6.70)	6.95 (6.20–7.98)	6.50 (5.80–7.65)	6.80 (5.80–7.50)	**<0.001**
Total cholesterol, mmol/L	6.97 (4.90–8.30)	4.40 (3.66–4.90)	4.50 (3.35–5.60)	5.30 (4.60–6.40)	**<0.001**

AIS, acute ischemic stroke; HS, hemorrhagic stroke; TIA, transient ischemic attack; NIHSS, National Institutes of Health Stroke Scale; n, number of participants. Continuous variables are presented as median (IQR), and categorical variables as n (%). Group comparisons were performed using the Kruskal–Wallis test for continuous variables and Pearson’s χ² test or Fisher’s exact test, as appropriate, for categorical variables. A two-sided P < 0.05 was considered statistically significant.

* Previous stroke was an exclusion criterion for participants in the control group.

Neurological deficit at admission, assessed using the NIHSS, differed significantly among the clinical groups (P < 0.001). Median NIHSS scores were 10.0 (7.25–14.75) in the HS group, 8.5 (4.0–13.0) in the AIS group, and 3.0 (3.0–6.0) in the TIA group. Post hoc pairwise comparisons using Dunn’s test with Holm–Bonferroni correction showed significant differences between TIA and AIS (P < 0.001) and between TIA and HS (P < 0.001), whereas AIS and HS did not differ significantly (P = 0.139).

On admission neuroimaging, no visible lesion was detected in 64.0% of patients with AIS, whereas right hemispheric involvement was observed in 52.0% of patients with HS. No focal brain lesions were identified in patients with TIA. According to the TOAST classification, 30 patients with AIS (60.0%) had large-artery atherosclerosis, 13 (26.0%) had cardioembolism, and 7 (14.0%) had stroke of undetermined etiology; no cases of small-vessel occlusion or stroke of other determined etiology were identified.

The prevalence of hypertension (P = 0.389), diabetes mellitus (P = 0.639), obesity (P = 0.110), previous myocardial infarction (P = 0.152), and chronic heart failure (P = 0.092) did not differ significantly among the groups. Atrial fibrillation differed significantly overall (P = 0.006), although no pairwise comparison remained significant after correction for multiple comparisons (all adjusted P > 0.05). Coronary artery disease also differed among the groups (P = 0.037), with a significant pairwise difference observed only between the TIA and control groups (adjusted P = 0.038). The prevalence of previous stroke differed across the four groups (P = 0.024); however, previous stroke was an exclusion criterion for control participants. When the analysis was restricted to the three clinical groups, no significant difference was observed (P = 0.284). Among participants with atrial fibrillation, anticoagulant therapy before hospital admission was reported in 11 of 13 (84.6%) patients with AIS, 1 of 4 (25.0%) with HS, 3 of 4 (75.0%) with TIA, and 2 of 3 (66.7%) controls.

Significant between-group differences were observed in leukocyte count, blood glucose, and total cholesterol (all P < 0.001), whereas hemoglobin and platelet count did not differ significantly (both P > 0.05). Post hoc pairwise comparisons using Dunn’s test with Holm–Bonferroni correction showed that leukocyte count differed significantly between HS and controls (P = 0.001) and between TIA and controls (P = 0.002). Blood glucose levels were higher in all three clinical groups than in controls (all P < 0.05), whereas total cholesterol levels were lower in the AIS and HS groups than in the TIA and control groups (all P < 0.05).

In the AIS group, 31 of 50 patients (62.0%) received intravenous thrombolysis with alteplase.

### Comparative analysis of circulating miRNA expression across the study groups

Before analysis of relative miRNA expression, Ct values of the endogenous reference miR-16-5p were evaluated. No significant differences were observed among the study groups at either sampling time point (S2 Table). Ct values of the exogenous spike-in control cel-miR-39 were consistent across samples (median, 26.76; IQR, 24.72–29.45), supporting stable technical performance of reverse transcription and subsequent amplification.

#### Comparative analysis of circulating miRNA expression at 3–4.5 h after symptom onset.

Table 2 presents the differences in circulating miRNA expression between the clinical groups and controls at 3–4.5 h after symptom onset. Significant differences were observed for let-7e-5p, miR-107, miR-125b-5p, and miR-222-3p. Let-7e-5p expression was significantly lower in all three clinical groups than in controls (all P < 0.001). A similar pattern was observed for miR-107, which was significantly downregulated in the AIS, HS, and TIA groups compared with controls (P = 0.012, 0.005, and 0.025, respectively). miR-125b-5p expression was significantly decreased in the AIS and TIA groups (P = 0.049 and 0.012, respectively), whereas the difference between HS and controls did not reach statistical significance (P = 0.054).

**Table 2:** Differential expression of circulating miRNAs in AIS, HS, and TIA compared with controls at 3–4.5 h after symptom onset.

miRNA	FC (AIS1 vs. control)	adjusted P value	FC (HS1 vs. control)	adjusted P value	FC (TIA1 vs. control)	adjusted P value
let-7e-5p	0.024	**<0.001**	0.033	**<0.001**	0.010	**<0.001**
miR-21-5p	0.589	0.647	0.661	0.454	0.244	0.067
miR-107	0.145	**0.012**	0.093	**0.005**	0.170	**0.025**
miR-125b-5p	0.061	**0.049**	0.146	0.054	0.042	**0.012**
miR-130a-3p	3.291	0.592	3.846	0.405	1.499	0.601
miR-143-3p	0.169	0.090	0.656	0.640	0.164	0.133
miR-222-3p	0.049	0.029	0.526	0.081	0.039	0.072

FC, fold change; AIS1, acute ischemic stroke at 3–4.5 h after symptom onset; HS1, hemorrhagic stroke at 3–4.5 h after symptom onset; TIA1, transient ischemic attack at 3–4.5 h after symptom onset. Fold change values were calculated using the 2^-ΔΔCt method and are presented relative to the сontrol group. Adjusted P values were obtained using the Holm–Bonferroni correction for multiple pairwise comparisons.

miR-222-3p expression was significantly decreased in the AIS group (P = 0.029), whereas no significant differences were observed between either the HS or TIA group and controls (both P > 0.05). No significant differences in miR-21-5p, miR-130a-3p, or miR-143-3p expression were observed between any of the clinical groups and controls (all P > 0.05).

#### Comparative analysis of circulating miRNA expression at 12 h after symptom onset.

At 12 h after symptom onset, significant differences in the expression of several circulating miRNAs were observed between the clinical groups and controls (Table 3). Let-7e-5p expression remained significantly lower in all three clinical groups than in controls (all P < 0.05). miR-107 expression remained significantly decreased in the AIS and HS groups (P = 0.006 and 0.002, respectively), whereas the difference between TIA and controls was not statistically significant (P = 0.064).

**Table 3:** Differential expression of circulating miRNAs in AIS, HS, and TIA compared with the controls at 12 h after symptom onset.

miRNA	FC (AIS2 vs. control)	adjusted P value	FC (HS2 vs. control)	adjusted P value	FC (TIA2 vs. control)	adjusted P value
let-7e-5p	0.020	**<0.001**	0.061	**0.002**	0.007	**<0.001**
miR-21-5p	0.935	0.732	1.067	0.721	0.163	0.057
miR-107	0.130	**0.006**	0.123	**0.002**	0.099	0.064
miR-125b-5p	0.044	**0.004**	0.245	0.080	0.054	0.054
miR-130a-3p	1.678	0.929	5.897	0.460	2.344	0.420
miR-143-3p	0.139	0.066	1.196	0.342	0.205	0.135
miR-222-3p	0.048	**0.027**	0.225	0.121	0.040	0.108

FC, fold change; AIS2, acute ischemic stroke at 12 h after symptom onset; HS2, hemorrhagic stroke at 12 h after symptom onset; TIA2, transient ischemic attack at 12 h after symptom onset. Fold change values were calculated using the 2^-ΔΔCt method relative to the сontrol group. P values were adjusted for multiple pairwise comparisons using the Holm–Bonferroni procedure.

miR-125b-5p expression was decreased in the AIS group (P = 0.004), whereas no differences were observed between HS or TIA and controls (P = 0.080 and 0.054, respectively). A similar pattern was observed for miR-222-3p, with lower expression detected only in AIS (P = 0.027). Expression of miR-21-5p, miR-130a-3p, and miR-143-3p did not differ between any of the clinical groups and controls (all P > 0.05).

No significant differences in circulating miRNA expression were observed between patients with AIS who received intravenous thrombolysis with alteplase and those who did not at either 3–4.5 h or 12 h after symptom onset after correction for multiple comparisons (all adjusted P > 0.05).

#### Intergroup differences in circulating miRNA expression among patients with AIS, HS, and TIA.

At 3–4.5 h after symptom onset, significant intergroup differences were observed for five of the seven investigated miRNAs (Fig 1), whereas miR-107 and miR-130a-3p did not differ among the clinical groups (all P > 0.05). let-7e-5p expression was lower in TIA than in AIS (median ΔCt, 8.03 vs. 6.75; FC = 2.43; P = 0.035) and HS (8.03 vs. 6.31; FC = 3.29; P = 0.022). Similarly, miR-21-5p expression was lower in TIA than in AIS (3.85 vs. 2.58; FC = 2.41; P = 0.002) and HS (3.85 vs. 2.42; FC = 2.69; P < 0.001). For miR-125b-5p, a difference was observed only between TIA and HS (8.14 vs. 6.34; FC = 3.48; P = 0.018), whereas TIA and AIS did not differ significantly. miR-143-3p and miR-222-3p showed higher expression in HS than in the other clinical groups. miR-143-3p expression was lower in both TIA (median ΔCt, 7.42 vs. 5.43; FC = 3.97; P = 0.003) and AIS (7.38 vs. 5.43; FC = 3.86; P = 0.003) than in HS. The corresponding differences for miR-222-3p were also observed between TIA and HS (8.59 vs. 6.30; FC = 4.89; P = 0.002) and between AIS and HS (8.27 vs. 6.30; FC = 3.92; P < 0.001).

**Fig 1:**
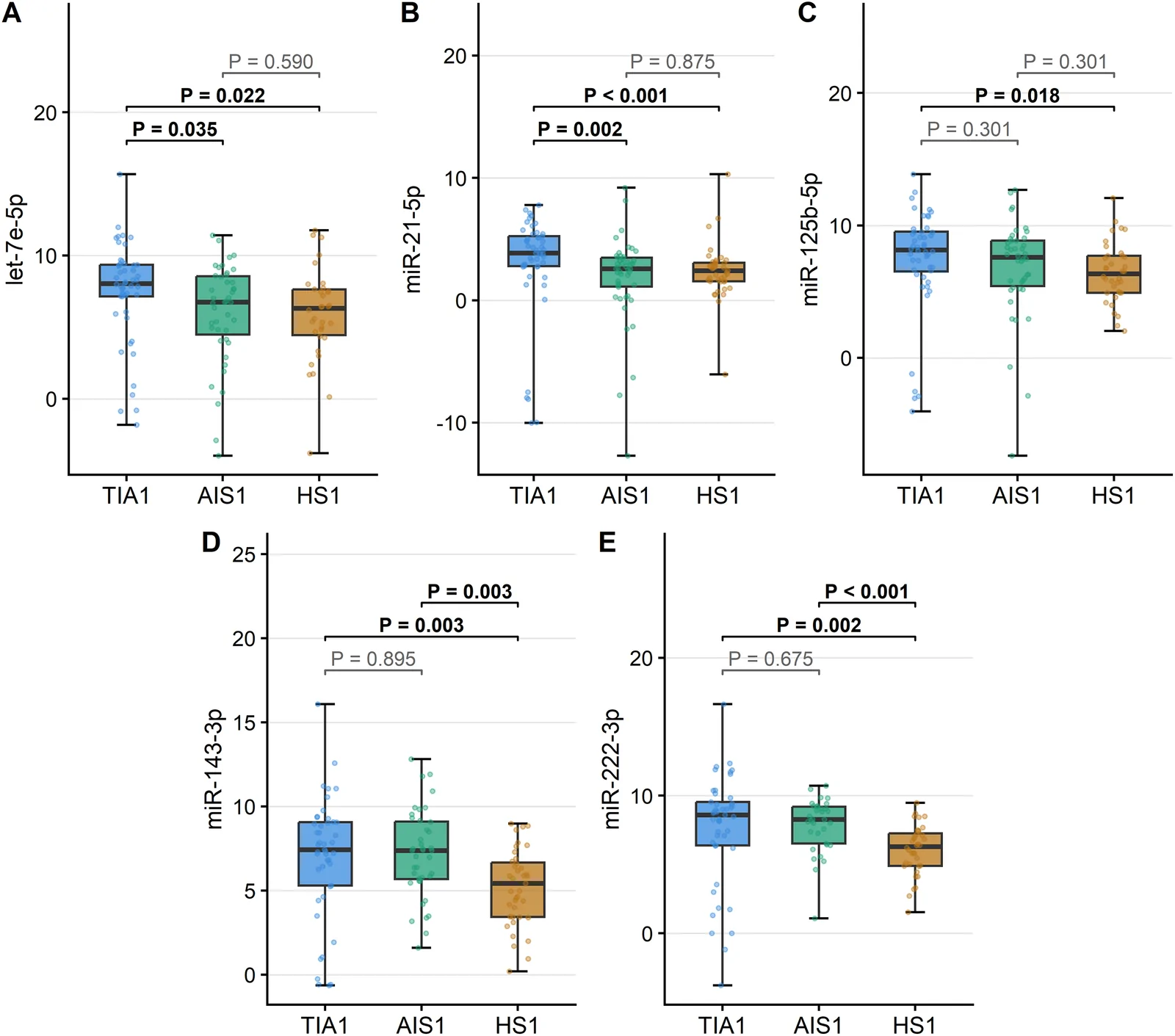
Intergroup differences in microRNA expression at 3–4.5 h after symptom onset. (A) let-7e-5p; (B) miR-21-5p; (C) miR-125b-5p; (D) miR-143-3p; (E) miR-222-3p. AIS1, acute ischemic stroke at 3–4.5 h after symptom onset; HS1, hemorrhagic stroke at 3–4.5 h after symptom onset; TIA1, transient ischemic attack at 3–4.5 h after symptom onset. Box plots show the median, interquartile range, minimum and maximum values; dots represent individual observations. Black indicates statistically significant pairwise differences after Holm–Bonferroni correction (P < 0.05), and gray indicates non-significant differences (P ≥ 0.05).

At 12 h after symptom onset, significant intergroup differences were observed for six of the seven investigated miRNAs (Fig 2), whereas miR-107 did not differ among the clinical groups (P > 0.05). let-7e-5p expression was lower in TIA and AIS than in HS (median ΔCt, 8.46 vs. 5.42; FC = 8.22; P = 0.001 and 7.03 vs. 5.42; FC = 3.05; P = 0.011, respectively). miR-21-5p expression was lower in TIA than in AIS (4.43 vs. 1.92; FC = 5.70; P < 0.001) and HS (4.43 vs. 1.72; FC = 6.54; P < 0.001). Lower miR-125b-5p expression was observed in both TIA (7.77 vs. 5.58; FC = 4.56; P = 0.009) and AIS (8.06 vs. 5.58; FC = 5.58; P < 0.001) compared with HS, whereas miR-130a-3p differed only between AIS and HS (7.52 vs. 5.70; FC = 3.53; P = 0.023). miR-143-3p and miR-222-3p again showed higher expression in HS than in AIS and TIA (all P < 0.001).

**Fig 2:**
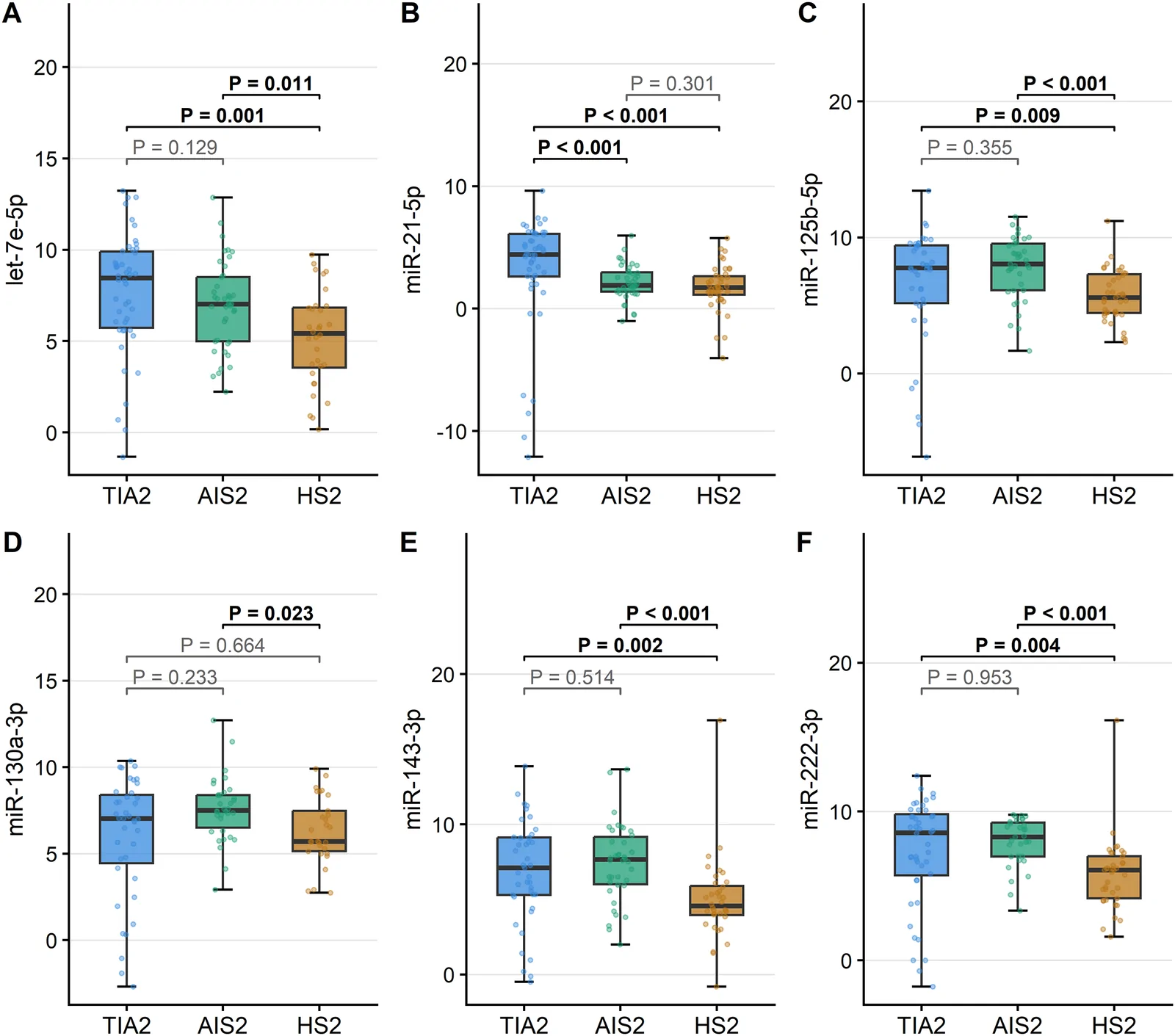
Intergroup differences in microRNA expression at 12 h after symptom onset. (A) let-7e-5p; (B) miR-21-5p; (C) miR-125b-5p; (D) miR-130a-3p; (E) miR-143-3p; (F) miR-222-3p. AIS2, acute ischemic stroke at 12 h after symptom onset; HS2, hemorrhagic stroke at 12 h after symptom onset; TIA2, transient ischemic attack at 12 h after symptom onset. Box plots show the median, interquartile range, minimum and maximum values; dots represent individual observations. Black indicates statistically significant pairwise differences after Holm–Bonferroni correction (P < 0.05), and gray indicates non-significant differences (P ≥ 0.05).

In the sensitivity analysis accounting for the broader multiple-testing burden across all 84 pairwise comparisons, all findings that remained significant after Holm–Bonferroni correction also retained statistical significance after Benjamini–Hochberg FDR adjustment.

#### Within-group changes in circulating miRNA expression during the first 12 h after symptom onset.

No significant changes in the expression of the investigated miRNAs were observed within any of the clinical groups between 3–4.5 h and 12 h after symptom onset (all P > 0.05, Wilcoxon signed-rank test).

#### miRNA expression according to TOAST classification.

Exploratory analysis according to TOAST classification revealed no significant differences in the expression of any of the investigated miRNAs among patients with large-artery atherosclerosis, cardioembolism, and stroke of undetermined etiology at either 3–4.5 h or 12 h after symptom onset (all P > 0.05, Kruskal–Wallis test).

#### Association of miRNA expression with functional outcome.

No associations between miRNA expression and mRS scores at discharge remained statistically significant after correction for multiple testing in either the AIS or HS group at either sampling time point. Likewise, miRNA expression did not differ between patients with favorable (mRS 0–2) and unfavorable (mRS 3–6) outcomes after FDR adjustment (all adjusted P > 0.05).

### Diagnostic performance of circulating miRNAs

#### ROC analysis of circulating miRNAs at 3–4.5 h after symptom onset.

At 3–4.5 h after symptom onset, let-7e-5p showed the highest AUC for discriminating AIS from controls (AUC = 0.788; 95% CI, 0.683–0.893; cutoff = 4.741; P < 0.001), with a sensitivity of 74.4% and specificity of 80.0%. miR-107 (AUC = 0.756; 95% CI, 0.592–0.921; cutoff = 5.129; P = 0.002), miR-125b-5p (AUC = 0.702; 95% CI, 0.551–0.852; cutoff = 5.054; P = 0.009), and miR-222-3p (AUC = 0.739; 95% CI, 0.548–0.930; cutoff = 5.091; P = 0.014) also showed significant discrimination between AIS and controls (Fig 3A).

**Fig 3:**
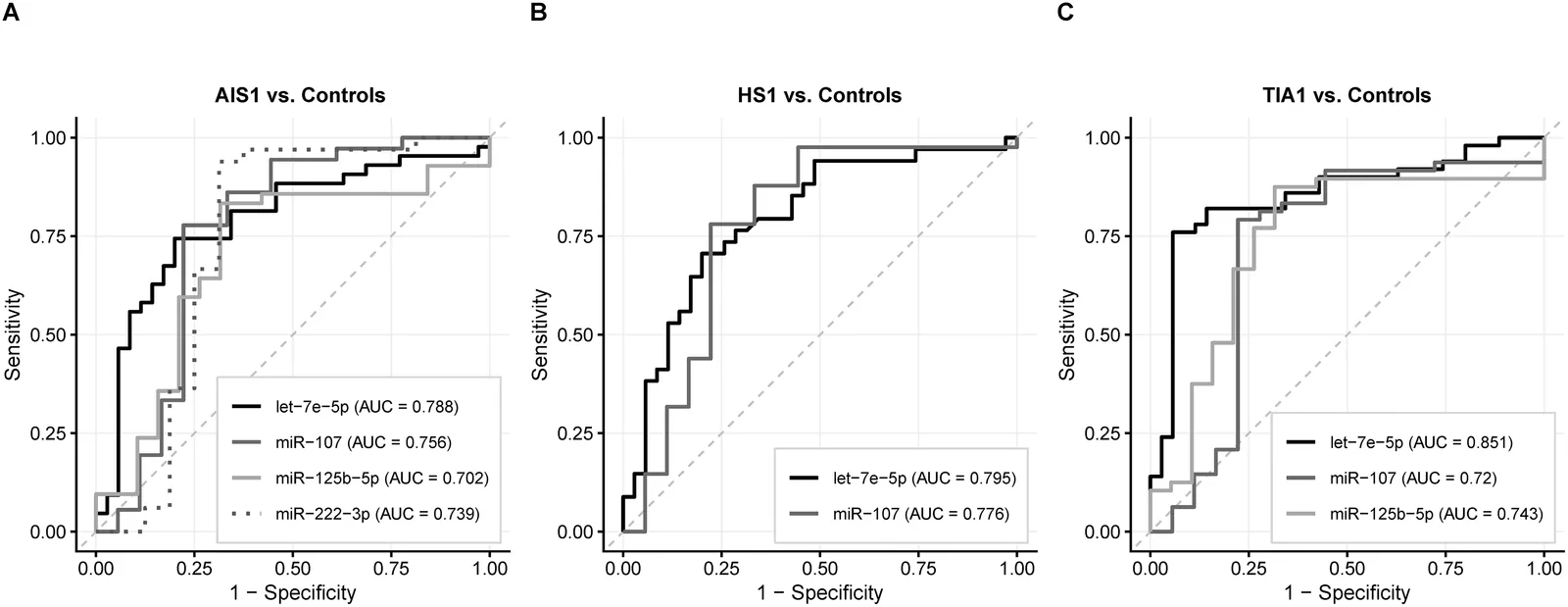
ROC curves of circulating miRNAs at 3–4.5 h after symptom onset. (A) AIS versus controls; (B) HS versus controls; (C) TIA versus controls. AIS1, acute ischemic stroke at 3–4.5 h after symptom onset; HS1, hemorrhagic stroke at 3–4.5 h after symptom onset; TIA1, transient ischemic attack at 3–4.5 h after symptom onset; AUC, area under the receiver operating characteristic curve; ROC, receiver operating characteristic. The legend shows the AUC for each miRNA. The dashed diagonal line represents random classification (AUC = 0.5).

For HS, the highest AUCs were observed for let-7e-5p (AUC = 0.795; 95% CI, 0.687–0.902; cutoff = 4.769; P < 0.001) and miR-107 (AUC = 0.776; 95% CI, 0.623–0.930; cutoff = 5.114; P < 0.001) (Fig 3B). miR-125b-5p showed a lower AUC of 0.696 (95% CI, 0.528–0.864; cutoff = 3.889; P = 0.022).

For TIA, let-7e-5p again showed the highest AUC (0.851; 95% CI, 0.764–0.939; cutoff = 7.029; P < 0.001), followed by miR-125b-5p (AUC = 0.743; 95% CI, 0.603–0.884; cutoff = 5.007; P = 0.001) and miR-107 (AUC = 0.720; 95% CI, 0.553–0.887; cutoff = 5.065; P = 0.010) (Fig 3C). Lower AUCs were observed for miR-21-5p (AUC = 0.676; P = 0.011) and miR-222-3p (AUC = 0.692; P = 0.024).

Overall, let-7e-5p yielded the highest AUC among the individual miRNAs for all three clinical groups at 3–4.5 h after symptom onset.

To assess the incremental diagnostic value of let-7e-5p, a clinical model incorporating age, sex, atrial fibrillation, blood glucose, and total cholesterol was evaluated. Analyses were restricted to participants with complete data for all model variables (n = 78; 39 AIS and 39 controls). The clinical model achieved an AUC of 0.832 (95% CI, 0.742–0.923), with a sensitivity of 92.3% and specificity of 64.1%. In the same subset, let-7e-5p alone yielded an AUC of 0.855 (95% CI, 0.767–0.942), with a sensitivity of 79.5% and specificity of 87.2%. Adding let-7e-5p to the clinical model increased the AUC to 0.982 (95% CI, 0.954–1.000), with both sensitivity and specificity of 94.9% (Fig 4). The AUC of the combined model was significantly higher than that of the clinical model (DeLong P < 0.001) and let-7e-5p alone (DeLong P = 0.002), whereas no difference was observed between the latter two (DeLong P = 0.750). Improvement in model fit after addition of let-7e-5p was also supported by the likelihood ratio test (χ² = 50.98, df = 1, P < 0.001). Internal validation using 1,000 bootstrap resamples yielded optimism-corrected AUCs of 0.789 for the clinical model and 0.957 for the combined model.

**Fig 4:**
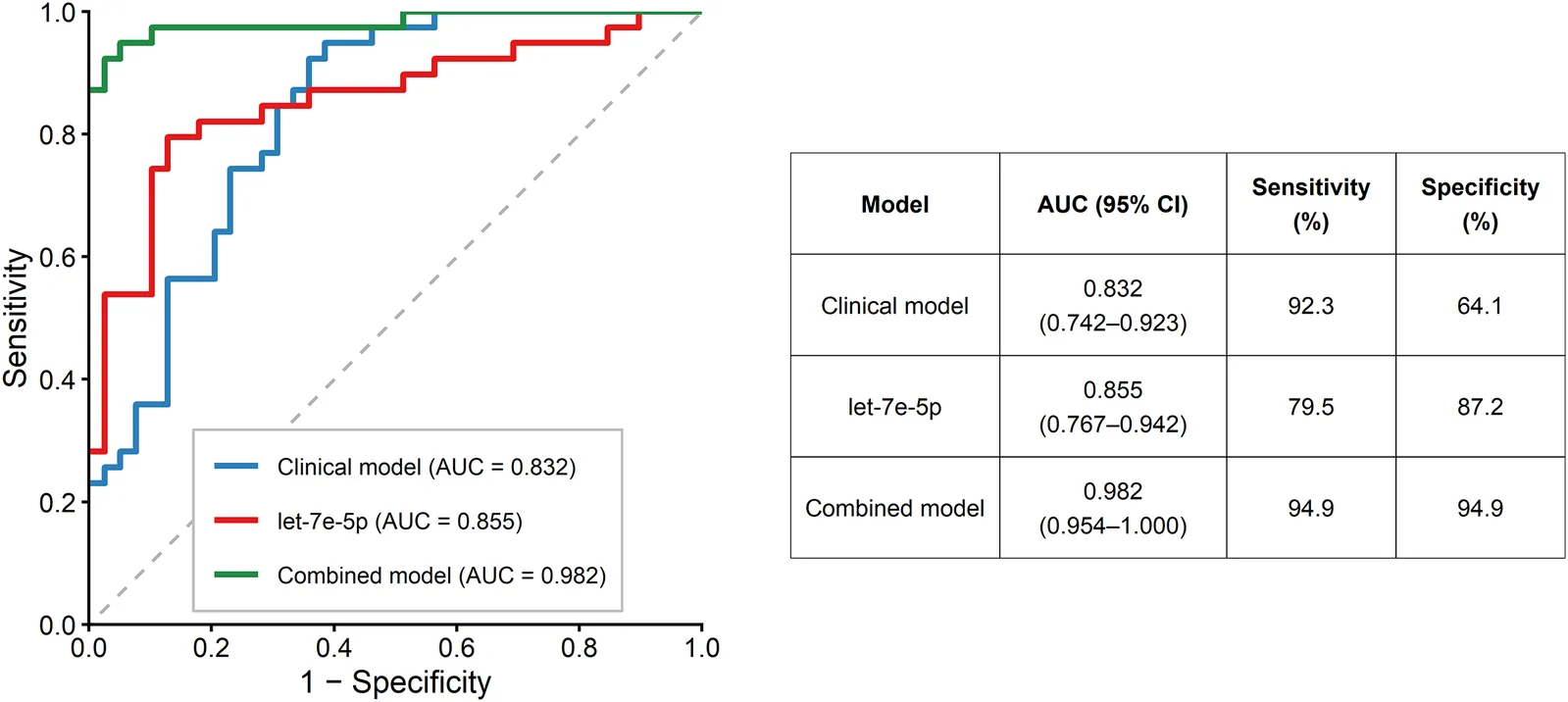
Diagnostic performance of clinical and miRNA-based models for discriminating AIS from controls.

ROC curves show the performance of the clinical model, let-7e-5p alone, and the combined model for discriminating AIS from controls at 3–4.5 h after symptom onset. The clinical model included age, sex, atrial fibrillation, blood glucose, and total cholesterol, whereas the combined model additionally included let-7e-5p. Analyses were performed in the common complete-case subset (n = 78; 39 AIS and 39 controls). The accompanying table presents AUCs with 95% CIs, sensitivity, and specificity for each model. Sensitivity and specificity were determined at the optimal cutoff using the Youden index. AUC, area under the receiver operating characteristic curve; AIS, acute ischemic stroke; CI, confidence interval; ROC, receiver operating characteristic.

To determine whether combining miRNAs improved discrimination, logistic regression models incorporating let-7e-5p and miR-107 were evaluated. For AIS, the two-miRNA model yielded an AUC of 0.777 (95% CI, 0.605–0.949), with an optimal cutoff of 0.657, sensitivity of 85.3%, and specificity of 71.4%. This was comparable to the AUC of let-7e-5p alone (0.788; 95% CI, 0.683–0.893). For HS, the two-miRNA model achieved an AUC of 0.818 (95% CI, 0.646–0.989), with an optimal cutoff of 0.507, sensitivity of 100.0%, and specificity of 64.3%. Although numerically higher than the AUC of let-7e-5p alone (0.795; 95% CI, 0.687–0.902), the difference was not statistically significant (DeLong Z = −0.726; P = 0.468).

#### ROC analysis of circulating miRNAs at 12 h after symptom onset.

At 12 h after symptom onset, let-7e-5p showed the highest AUC for discriminating AIS from controls (AUC = 0.867; 95% CI, 0.782–0.952; cutoff = 4.785; P < 0.001). miR-125b-5p (AUC = 0.774; 95% CI, 0.623–0.925; cutoff = 5.033; P < 0.001), miR-107 (AUC = 0.768; 95% CI, 0.597–0.938; cutoff = 5.006; P = 0.002), and miR-222-3p (AUC = 0.744; 95% CI, 0.550–0.938; cutoff = 5.125; P = 0.014) also showed significant discrimination between AIS and controls (Fig 5A).

**Fig 5:**
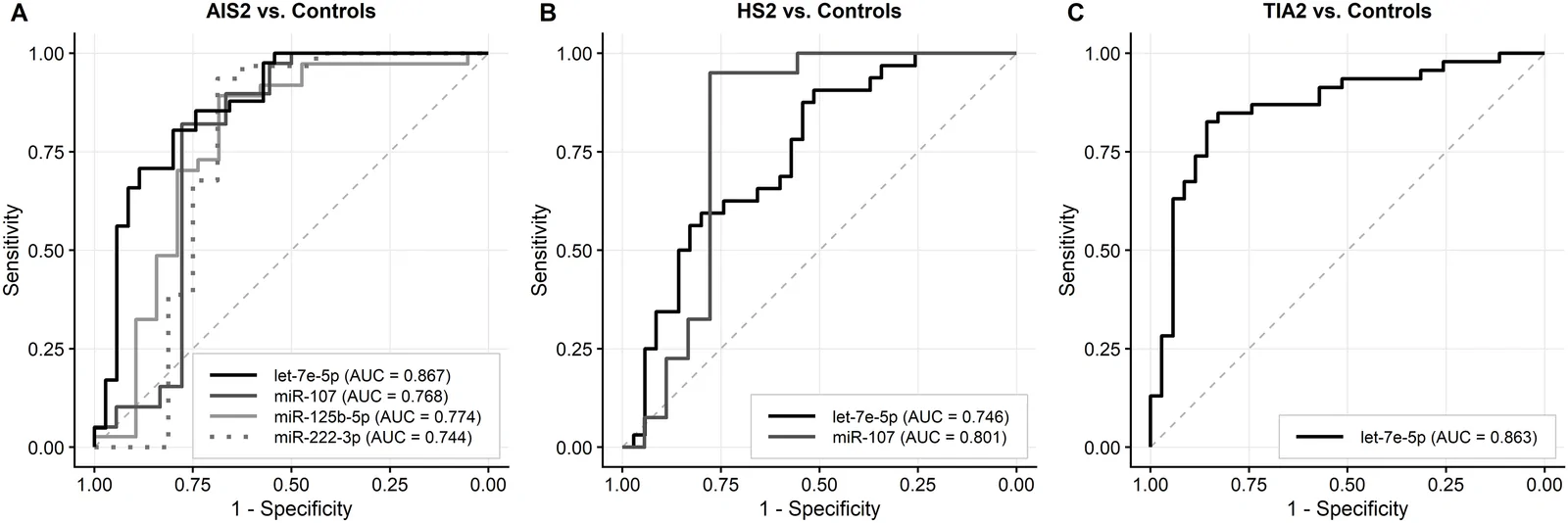
ROC curves of circulating miRNAs at 12 h after symptom onset. (A) AIS versus controls; (B) HS versus controls; (C) TIA versus controls. The legend shows the AUC for each miRNA. AIS2, acute ischemic stroke at 12 h after symptom onset; HS2, hemorrhagic stroke at 12 h after symptom onset; TIA2, transient ischemic attack at 12 h after symptom onset; AUC, area under the receiver operating characteristic curve; ROC, receiver operating characteristic. The dashed diagonal line represents random classification (AUC = 0.5).

For HS, the highest AUC was observed for miR-107 (AUC = 0.801; 95% CI, 0.637–0.966; cutoff = 5.007; P < 0.001), followed by let-7e-5p (AUC = 0.746; 95% CI, 0.629–0.864; cutoff = 1.501; P < 0.001) (Fig 5B).

For TIA, let-7e-5p yielded the highest AUC (0.863; 95% CI, 0.779–0.948; cutoff = 5.525; P < 0.001) (Fig 5C). Although the ROC analyses for miR-21-5p, miR-107, miR-125b-5p, and miR-222-3p were statistically significant, their AUCs did not exceed 0.700.

Overall, let-7e-5p yielded the highest AUC among the individual miRNAs for discriminating AIS and TIA from controls, whereas miR-107 showed the highest AUC for HS.

A combined diagnostic model incorporating let-7e-5p, miR-107, and miR-125b-5p was additionally constructed for AIS. The model yielded an AUC of 0.888 (95% CI, 0.759–1.000), with an optimal cutoff of 0.624, a sensitivity of 91.2%, and a specificity of 81.8%. Although the AUC was numerically higher than that of let-7e-5p alone (AUC = 0.867; 95% CI, 0.782–0.952), the difference was not statistically significant according to DeLong’s test (Z = −1.002; P = 0.317).

### ROC analysis for discrimination between clinical groups

ROC analysis was performed to assess the ability of the investigated miRNAs to distinguish between AIS, HS, and TIA using pairwise comparisons of the clinical groups. Only miRNAs showing significant intergroup differences in the preceding comparative analysis were included. Among these, miR-222-3p, miR-21-5p, and miR-143-3p showed the highest AUCs.

The results of the ROC analysis are summarized in Table 4. Among the investigated miRNAs, miR-222-3p yielded the highest AUC for differentiating the clinical subtypes of acute cerebrovascular disease. The best performance was observed for discrimination between AIS and HS at 12 h after symptom onset (AUC = 0.833; 95% CI, 0.730–0.935), as illustrated by the ROC curve in Fig 6. miR-143-3p also demonstrated favorable discrimination between these two groups, with a maximum AUC of 0.785. In contrast, miR-21-5p performed best in differentiating TIA from stroke, achieving a maximum AUC of 0.767.

**Table 4:** ROC analysis of circulating miRNAs for pairwise discrimination between clinical groups.

miRNA	Comparison	AUC (95% CI)	Cutoff	Sensitivity, %	Specificity, %
**miR-222-3p**	AIS1–HS1	0.774 (0.661–0.888)	7.54	83.3	66.7
	TIA1–HS1	0.716 (0.598–0.834)	7.92	86.1	62.2
	AIS2–HS2	0.833 (0.730–0.935)	7.72	94.6	67.7
	TIA2–HS2	0.702 (0.580–0.823)	7.74	94.6	54.5
**miR-21-5p**	AIS1–TIA1	0.697 (0.587–0.806)	3.71	57.1	82.6
	HS1–TIA1	0.723 (0.611–0.835)	3.35	63.3	85.4
	AIS2–TIA2	0.756 (0.649–0.863)	4.23	58.3	95.1
	HS2–TIA2	0.767 (0.663–0.872)	3.36	66.7	88.4
**miR-143-3p**	AIS1–HS1	0.710 (0.596–0.824)	6.94	79.5	57.5
	HS1–TIA1	0.707 (0.592–0.822)	6.76	76.9	66.7
	AIS2–HS2	0.785 (0.675–0.895)	6.15	80.6	73.7
	HS2–TIA2	0.705 (0.582–0.829)	7.22	92.1	51.3

AIS1, acute ischemic stroke at 3–4.5 h after symptom onset; AIS2, acute ischemic stroke at 12 h after symptom onset; HS1, hemorrhagic stroke at 3–4.5 h after symptom onset; HS2, hemorrhagic stroke at 12 h after symptom onset; TIA1, transient ischemic attack at 3–4.5 h after symptom onset; TIA2, transient ischemic attack at 12 h after symptom onset; AUC, area under curve; CI, confidence interval. Cutoff values were determined using the Youden index.

**Fig 6:**
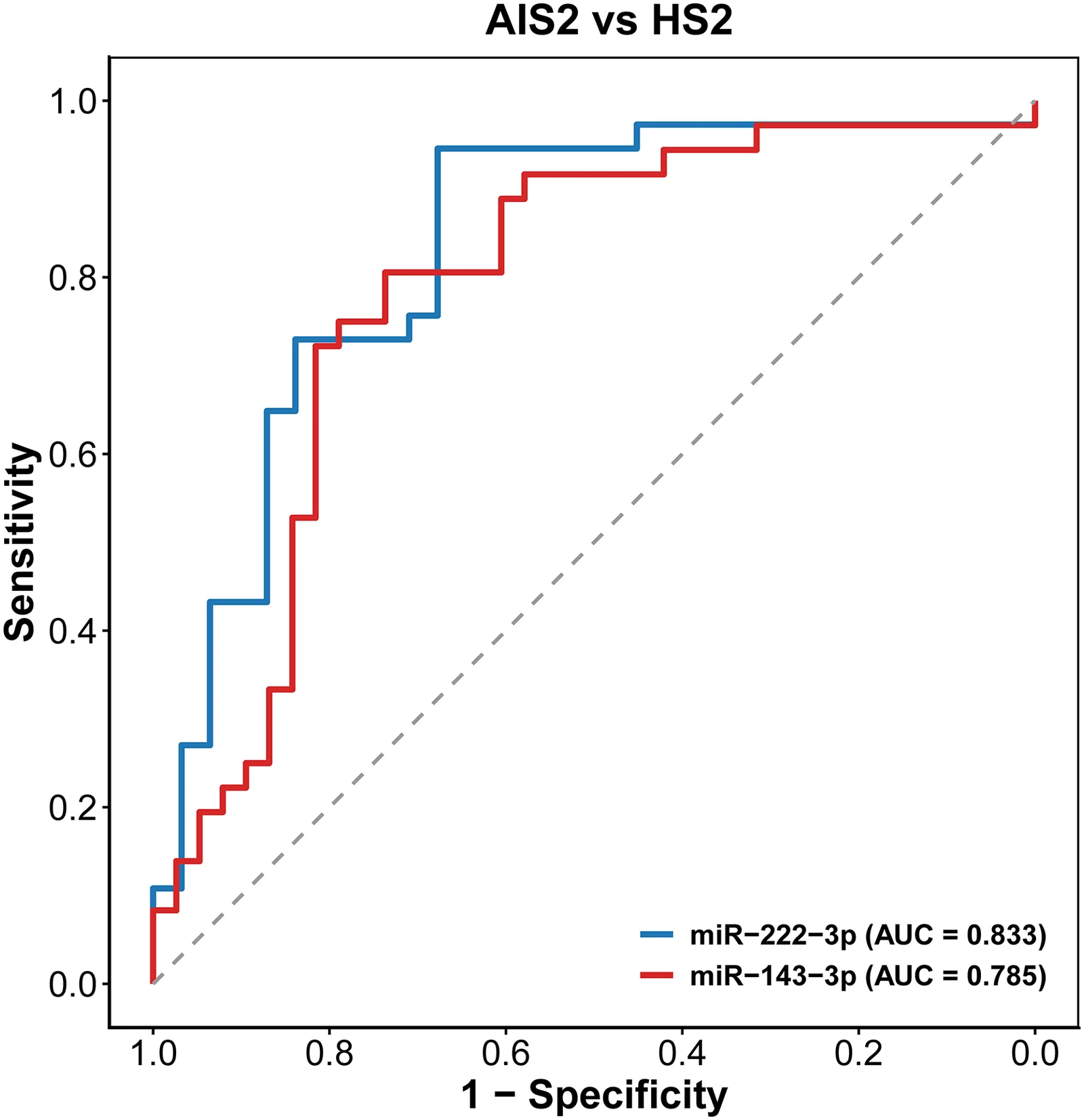
ROC curves of miR-222-3p and miR-143-3p for discriminating AIS from HS at 12h. AUC values for each microRNA are shown in the legend. AIS2, acute ischemic stroke at 12 h after symptom onset; HS2, hemorrhagic stroke at 12 h after symptom onset; AUC, area under the receiver operating characteristic curve; ROC, receiver operating characteristic. The dashed diagonal line represents random classification (AUC = 0.5).

To assess whether combining miRNAs improved discrimination between AIS and HS, a logistic regression model incorporating miR-222-3p and miR-143-3p was evaluated using samples collected at 12 h after symptom onset. The combined model yielded an AUC of 0.857 (95% CI, 0.759–0.956), with an optimal probability cutoff of 0.518, sensitivity of 81.3%, and specificity of 80.0%. Adding miR-143-3p to miR-222-3p significantly improved model fit (χ² = 4.38; P = 0.036). However, the increase in AUC compared with miR-222-3p alone (0.857 vs. 0.833) was not statistically significant (DeLong Z = −0.993; P = 0.321).

As miR-222-3p showed the highest individual AUC for discriminating AIS from HS at 12 h, its performance was further evaluated using 1,000 bootstrap resamples. The apparent AUC was 0.833, with an estimated optimism of 0.001, yielding an optimism-corrected AUC of 0.832.

### Correlations between miRNA expression and clinical, demographic, and laboratory characteristics

Before correction for multiple testing, several nominal associations were observed between miRNA expression and the investigated characteristics. In the AIS group, miR-107 expression was inversely correlated with leukocyte count at both 3–4.5 h (r = −0.343; P = 0.041) and 12 h (r = −0.396; P = 0.013). In the HS group, an inverse association was found between let-7e-5p expression and platelet count at 12 h (r = −0.424; P = 0.016), while in the TIA group, let-7e-5p expression was positively associated with age at 12 h (r = 0.327; P = 0.026). None of these associations remained statistically significant after Benjamini–Hochberg FDR correction (all adjusted P > 0.05).

## Discussion

In this exploratory case-control study, we evaluated the circulating expression and diagnostic performance of seven candidate miRNAs in patients with AIS, HS, and TIA at two time points within the first 12 h after symptom onset. Several miRNAs exhibited distinct expression patterns across the diagnostic groups. Among the investigated markers, let-7e-5p yielded the highest individual AUCs for comparisons of AIS and TIA with controls, whereas miR-222-3p showed the strongest individual discrimination between AIS and HS. Multi-miRNA models did not significantly outperform the corresponding individual markers. By contrast, the addition of let-7e-5p to a model comprising routinely available clinical variables improved discrimination between AIS and controls.

Beyond differences in miRNA expression, the study groups exhibited distinct clinical and laboratory profiles. Patients with AIS and HS presented with greater neurological impairment than those with TIA, as reflected by their NIHSS scores. Blood glucose concentrations were higher in the clinical groups than in controls, consistent with hyperglycemia commonly observed during the acute phase of AIS and potentially reflecting, at least in part, a stress-related metabolic response to cerebral injury [30]. Conversely, total cholesterol concentrations were lower in AIS and HS than in controls. The relationship between total cholesterol and cerebrovascular disease remains complex, as recent evidence links higher cholesterol concentrations to increased stroke risk while also demonstrating considerable inconsistency across studies [31].

Among the investigated miRNAs, let-7e-5p showed the most consistent expression pattern, with lower levels in AIS, HS, and TIA than in controls at both sampling time points. It also yielded the highest individual AUCs for discriminating AIS and TIA from controls at 12 h. This finding is consistent with accumulating evidence implicating circulating let-7e-5p in ischemic stroke. A meta-analysis of 29 studies evaluating 112 circulating miRNAs identified differential expression of let-7e-5p, which was subsequently confirmed in an independent plasma cohort, where it discriminated ischemic stroke from healthy controls with an AUC of 0.751. Target-gene enrichment in the same study further linked the validated miRNAs to neuroinflammatory and cellular-senescence pathways [32]. A previous systematic review likewise identified let-7e among circulating miRNAs associated with different etiological subtypes of AIS; however, its direction of dysregulation was inconsistent across studies, with both increased and decreased expression reported relative to healthy controls **[27]**. More recently, an umbrella review encompassing 220 miRNAs highlighted let-7e-5p among circulating markers with relatively consistent evidence in AIS and emphasized temporal and methodological variability as major challenges to biomarker translation, particularly with respect to sampling windows and assay standardization [33]. Such heterogeneity is also evident in recent primary data: in a cohort of 60 patients with AIS and 30 age- and sex-matched controls, whole-blood let-7e-5p levels did not differ at hospital admission but declined by day 7 (P < 0.001) [34]. Collectively, the available evidence indicates recurrent alterations in let-7e-5p in cerebrovascular disease, while suggesting that their direction and magnitude may depend on sampling time, biological matrix, and analytical methodology.

miR-107 also exhibited a relatively consistent pattern, with lower levels in AIS and HS than in controls at both sampling time points, whereas the difference in TIA was confined to the early assessment. Its diagnostic performance was moderate, with the highest AUC observed for HS versus controls at 12 h (AUC = 0.801), while no differences were detected among the clinical groups. Previous evidence similarly suggests sustained miR-107 dysregulation following ischemic stroke. In a longitudinal study evaluating 754 plasma miRNAs, miR-107 was consistently reduced in patients with ischemic stroke compared with matched controls at 5, 15, and 30 days after the event, with the decrease remaining significant after correction for multiple testing across all study time points [28]. A more recent hybrid umbrella review of early-phase fluid biomarkers in AIS also identified miR-107 among circulating miRNAs with relatively consistent evidence of dysregulation [33]. Complementary bioinformatic evidence has implicated miR-107 in a regulatory network shared between type 2 diabetes mellitus and cardiogenic stroke, together with miR-34a-5p, miR-103-3p, and miR-124-3p; the predicted network involved genes related to immune responses and other pathophysiological processes [35]. The reduction observed in the present cohort, together with alterations reported at later stages of ischemic stroke, suggests that changes in circulating miR-107 may occur across different phases of the disease. However, the absence of differences among AIS, HS, and TIA in our cohort indicates that its value for distinguishing between specific acute cerebrovascular conditions remains uncertain.

miR-125b-5p showed a less consistent and predominantly AIS-related pattern. Its levels were lower in AIS than in controls at both sampling time points, whereas the difference in TIA was confined to 3–4.5 h and no differences were observed in HS. Its highest AUC was observed for AIS at 12 h (AUC = 0.774). Systematic reviews have repeatedly implicated miR-125b-5p in AIS, although the reported direction of dysregulation has varied. One review identified miR-125b-5p among circulating miRNAs reported to be upregulated in AIS [9], whereas another focusing specifically on the first 12 h after stroke onset reported altered miR-125b-5p expression with subsequent normalization by 90 days and emphasized substantial heterogeneity in sampling and the lack of independent validation across the included studies [36]. A more recent systematic review of cell-free nucleic acids also identified miR-125b-5p among miRNAs with replicated diagnostic evidence, although independent validation remained uncommon across the field [37]. These observations are compatible with an association between circulating miR-125b-5p and AIS, but the variability across studies limits conclusions regarding a consistent direction of expression or diagnostic utility.

miR-222-3p exhibited a distinct pattern, characterized by lower levels in AIS than in controls at both sampling time points and higher levels in HS than in AIS and TIA. Among the miRNAs examined, it provided the strongest individual discrimination between AIS and HS, with AUCs of 0.774 at 3–4.5 h and 0.833 at 12 h. Internal bootstrap assessment of the 12-h model yielded an optimism-corrected AUC of 0.832, suggesting little optimism in the apparent estimate. Previous evidence also indicates that circulating miR-222-3p may be altered during the course of ischemic stroke. In a study evaluating serum exosomal miRNAs after reperfusion therapy, miR-222-3p levels decreased between days 1 and 10 in patients who subsequently had unfavorable functional outcomes at 90 days, with a similar reduction observed among patients receiving combined intravenous thrombolysis and mechanical thrombectomy [38]. Complementary bioinformatic evidence identified miR-222-3p, together with miR-221-3p, as a potential regulator within molecular networks shared by cardioembolic stroke and type 2 diabetes mellitus [39]. Although these studies differ in biological material, sampling time, and clinical context, they provide complementary evidence linking miR-222-3p to ischemic cerebrovascular disease. In the present cohort, its most notable feature was discrimination between AIS and HS, suggesting that further investigation may be particularly relevant to differentiation between ischemic and hemorrhagic stroke.

In contrast, miR-21-5p, miR-130a-3p, and miR-143-3p did not differ from controls at either sampling time point but exhibited distinct patterns across AIS, HS, and TIA. miR-21-5p was lower in TIA than in both AIS and HS, with the strongest discrimination observed between HS and TIA at 12 h (AUC = 0.767). Experimental evidence provides a biological context for the involvement of miR-21-5p in cerebral ischemia: hypoxia induced rapid secretion of extracellular vesicle-associated miR-21a-5p from neuronal cells, while higher circulating miR-21-5p levels in patients with stroke were associated with greater disability at hospital discharge [40]. miR-130a-3p showed a more restricted pattern, differing only between AIS and HS at 12 h. This contrasts with a study of 85 patients with AIS and 85 healthy controls, in which plasma miR-130a-3p was elevated in AIS and demonstrated an AUC of 0.831; its levels were also associated with glucose, inflammatory markers, and stroke severity [41]. miR-143-3p showed more consistent differences among the clinical groups, with higher levels in HS than in AIS and TIA at both sampling time points and moderate discrimination between AIS and HS, reaching an AUC of 0.785 at 12 h. A systematic review of biomarkers for early stroke detection identified miR-143-3p among circulating miRNAs reported to be upregulated in AIS [9]. The observed patterns suggest that miR-21-5p, miR-130a-3p, and miR-143-3p may reflect differences within the spectrum of acute cerebrovascular disease that are less apparent in comparisons with controls, although their expression appears to vary across clinical and methodological contexts.

Combining multiple miRNAs did not significantly improve discrimination over the best-performing individual markers. Although modest numerical increases in AUC were observed for some multi-miRNA models, these gains were not significant by DeLong’s test. Notably, adding miR-143-3p to miR-222-3p improved model fit for AIS–HS discrimination despite the absence of a significant increase in AUC. In a recent systematic review and meta-analysis of blood biomarkers for ischemic stroke, studies evaluating multiple biomarkers showed a higher pooled AUC than those evaluating single biomarkers (0.91 vs. 0.88), although substantial heterogeneity was observed across study characteristics [42]. The potential advantage of multi-biomarker approaches may therefore depend less on the number of included markers than on whether additional variables contribute discriminatory information beyond that captured by the strongest individual marker.

A different pattern emerged when molecular information was integrated with clinical variables. Adding let-7e-5p to the model comprising age, sex, atrial fibrillation, blood glucose, and total cholesterol increased the AUC for AIS versus controls to 0.982 and outperformed both the clinical model and let-7e-5p alone; discrimination remained high after internal bootstrap correction. This observation is consistent with recent evidence examining the integration of blood biomarkers with clinical information. A systematic review of blood-based biomarkers measured within 12 h of AIS onset found that multi-marker approaches, particularly those incorporating biomarkers with clinical scales, achieved some of the highest reported diagnostic performance, including an AUC of 0.95 for a model combining D-dimer and glial fibrillary acidic protein (GFAP) with the Field Assessment Stroke Triage for Emergency Destination (FAST-ED) [43]. Although these findings arise from different biomarkers and study designs, they provide a rationale for evaluating whether molecular measurements add information to routinely available clinical variables rather than considering them solely as stand-alone tests.

None of the investigated miRNAs showed significant within-group changes between 3–4.5 h and 12 h after symptom onset, suggesting relative stability over this early interval. Evidence on the temporal behavior of circulating miRNAs during the hyperacute phase of stroke remains limited. A recent systematic review of miRNAs measured within the first 12 h of AIS found that only two of six included studies evaluated temporal expression, with heterogeneity in sampling times precluding quantitative synthesis [36]. Nevertheless, temporal profiles appear to be miRNA-specific. In a longitudinal study of patients with acute large-vessel occlusion stroke, miR-140-5p and miR-7-5p were elevated within the first 6 h and subsequently declined toward control levels, whereas miR-210-3p showed an inverse pattern over the following 12–24 h [44]. The absence of detectable changes in our cohort should therefore be interpreted as a lack of evidence for temporal variation over the interval examined, rather than evidence that these miRNAs remain stable throughout the course of stroke.

Exploratory analysis according to TOAST classification revealed no differences in the investigated miRNAs among patients with large-artery atherosclerosis, cardioembolism, and stroke of undetermined etiology at either sampling time point. Previous studies, however, have reported circulating miRNA alterations associated with AIS etiology. A systematic review of 12 studies identified several potentially subtype-associated miRNAs, although replication across studies was limited, with only let-7b and let-7e repeatedly associated with cardioembolic stroke [27]. Similarly, no associations between miRNA expression and functional outcome at discharge remained significant after correction for multiple testing in either AIS or HS. In contrast, a recent study profiling plasma miRNAs within 24 h of ischemic stroke identified five miRNAs whose associations with poor functional outcome at 3 months were confirmed in an independent replication cohort [45]. Associations between miRNA expression and other clinical or laboratory variables in our cohort were likewise limited to nominal correlations that did not remain significant after multiple-testing correction. Overall, these exploratory analyses provided limited evidence of robust associations between the investigated miRNAs and AIS etiology, clinical characteristics, or functional outcome.

The present study has several strengths. It provides a comparative evaluation of circulating miRNAs across AIS, HS, and TIA using a standardized protocol for RNA isolation and miRNA quantification. Sampling at two predefined early time points (3–4.5 h and 12 h after symptom onset) enabled assessment of miRNA expression during the early phase of cerebrovascular disease. Beyond individual miRNA performance, the study examined whether combining miRNAs or integrating molecular and routinely available clinical information provided additional discriminatory value. The use of formal model comparisons and internal bootstrap validation further strengthened the assessment of diagnostic performance.

Several limitations should be acknowledged. This exploratory case-control study was conducted at two stroke centers within a single geographic region, which may limit the generalizability of the findings. Importantly, the control group consisted of stable ambulatory patients with hypertension rather than individuals presenting with acute neurological symptoms or stroke-mimicking conditions. The resulting contrast between established cerebrovascular disease and clinically stable controls may have introduced spectrum bias and produced optimistic estimates of diagnostic performance relative to those expected in an unselected acute-care population. This concern is particularly relevant given a systematic review and meta-analysis including patients with ischemic stroke, HS, stroke mimics, and healthy controls, in which no investigated protein or non-coding RNA biomarker demonstrated high diagnostic performance for distinguishing stroke from stroke mimics [46]. Accordingly, the AUCs, sensitivity, specificity, and derived cut-off values reported here should be considered exploratory and require prospective evaluation in consecutive patients presenting with suspected acute stroke, including clinically relevant stroke mimics. Differences in cardiovascular comorbidities among the study groups may also have influenced circulating miRNA profiles and cannot be fully separated from effects related to the acute cerebrovascular event.

The sample size also limited several secondary analyses, particularly those stratified by AIS etiology and functional outcome. Eligibility was restricted to patients with NIHSS scores of 3–23, resulting in underrepresentation of very mild and very severe presentations and limiting extrapolation across the full spectrum of stroke severity. Furthermore, the two sampling points covered only the first 12 h after symptom onset and therefore cannot characterize miRNA trajectories beyond the early phase. The same control group served as the reference for both sampling time points; comparisons with controls at 3–4.5 h and 12 h consequently represent repeated comparisons with a common reference rather than independent replication. Quantitative infarct-volume data were not systematically available, precluding evaluation of relationships between miRNA expression and lesion volume. Functional outcome was assessed at discharge rather than at a standardized longer-term time point such as 90 days, limiting conclusions regarding prognostic associations.

miR-16-5p, which may be sensitive to hemolysis, was used as the sole endogenous reference. Although hemolyzed samples were excluded and miR-16-5p Ct values showed limited variability without differences among groups, multiple candidate reference miRNAs were not evaluated using stability algorithms such as geNorm, NormFinder, or BestKeeper; residual normalization bias therefore cannot be excluded. In addition, although internal bootstrap procedures were used to estimate optimism for specific diagnostic models, the findings were not externally validated. Independent validation in larger, preferably multicenter prospective cohorts will be necessary to determine the stability of model performance and derived thresholds across populations and clinical settings.

Finally, the laboratory-based RT-qPCR workflow used in the present study requires multiple processing and analytical steps, limiting its applicability to immediate decision-making within the time-sensitive reperfusion pathway. Circulating miRNA measurements should therefore be viewed, at present, as a potential complement to established clinical assessment and emergency neuroimaging rather than as a replacement for these approaches. Future studies should prioritize rapid and standardized analytical platforms and prospectively evaluate candidate miRNAs in unselected patients with suspected acute stroke to determine whether they provide clinically meaningful incremental diagnostic value under real-world conditions.

## Conclusion

In this exploratory case-control study, circulating miRNAs showed distinct expression and discrimination patterns across AIS, HS, TIA, and controls during the first 12 h after symptom onset. Among the investigated miRNAs, let-7e-5p showed the most consistent differences between the clinical groups and controls, whereas miR-222-3p provided the strongest individual discrimination between AIS and HS. Combining multiple miRNAs did not significantly improve discrimination over the best-performing individual markers, while integration of let-7e-5p with routinely available clinical variables improved discrimination between AIS and controls. These findings provide a basis for further investigation of circulating miRNAs as complementary diagnostic markers in acute cerebrovascular disease. Prospective validation in larger, independent cohorts that include patients with stroke-mimicking conditions is needed to determine their incremental value and clinical applicability in real-world acute stroke assessment.

## Supporting information

S1 Table TaqMan™ Advanced miRNA Assays used in the study. (DOCX)

S2 Table Ct values of the endogenous reference miRNA miR-16-5p in the study groups. AIS: acute ischemic stroke, HS: hemorrhagic stroke, TIA: transient ischemic attack, h: hours. Data are presented as median (interquartile range, IQR). P-values were calculated using the Kruskal–Wallis test. The first time point corresponds to blood sampling performed 3–4.5 h after symptom onset, and the second time point to 12 h after symptom onset.
(DOCX)
