# Supplementary material for: Diagnostic performance of circulating microRNAs for the early diagnosis of different types of acute cerebrovascular disease
Source: PLoS One. 2026 Oct 5;21(10):e0359648. doi: 10.1371/journal.pone.0359648 (PMC13637907; doi:10.1371/journal.pone.0359648)
Supplement: Supplementary file 1 [file pone.0359648.s001.docx]

**S1 Table**. TaqMan™ Advanced miRNA Assays used in the study

| **microRNA** | **Assay ID** |
| --- | --- |
| hsa-let-7e-5p | 478579_mir |
| hsa-miR-16-5p | 477860_mir |
| hsa-miR-21-5p | 477975_mir |
| hsa-miR-107 | 478254_mir |
| hsa-miR-125b-5p | 477885_mir |
| hsa-miR-130a-3p | 477851_mir |
| hsa-miR-143-3p | 477912_mir |
| hsa-miR-222-3p | 477982_mir |
| cel-miR-39-3p | 478293_mir |
