# Supplementary material for: Diagnostic performance of circulating microRNAs for the early diagnosis of different types of acute cerebrovascular disease
Source: PLoS One. 2026 Oct 5;21(10):e0359648. doi: 10.1371/journal.pone.0359648 (PMC13637907; doi:10.1371/journal.pone.0359648)
Supplement: Supplementary file 2 [file pone.0359648.s002.docx]

**S2 Table.** Ct values of the endogenous reference miRNA miR-16-5p in the study groups.

| **Time point** | **Controls** | **AIS** | **HS** | **TIA** | **p-value** |
| --- | --- | --- | --- | --- | --- |
| **3–4.5 h** | 22,54 (21,98–23,11) | 22.63 (22.10–23.35) | 22.71 (22.08–23.40) | 22,58 (22,01–23,18) | 0.72 |
| **12 h** | 22,54 (21,98–23,11) | 22.68 (22.05–23.29) | 22.74 (22.14–23.36) | 22.60 (22.03–23.20) | 0.81 |

AIS: acute ischemic stroke, HS: hemorrhagic stroke, TIA: transient ischemic attack, h: hours. Data are presented as median (interquartile range, IQR). P-values were calculated using the Kruskal–Wallis test. The first time point corresponds to blood sampling performed 3–4.5 h after symptom onset, and the second time point to 12 h after symptom onset.
