# Supplementary material for: Diagnostic performance of circulating microRNAs for the early diagnosis of different types of acute cerebrovascular disease
Source: PLoS One. 2026 Oct 5;21(10):e0359648. doi: 10.1371/journal.pone.0359648 (PMC13637907; doi:10.1371/journal.pone.0359648)
Supplement: Supplementary file 3 [file pone.0359648.s003.docx]

Thanks for invitation to review this manuscript. Authors studied an important clinical problem to identify blood based biomarkers for early diagnosis of AIS, HS, TIA in comparison with the healthy control samples. This is a prospective study (includes 50 sample in each group) evaluating seven biologically plausible microRNAs using RT-qPCR.

**Comments for Authors:**

Although the topic is timely and clinically relevant, several methodological, statistical, and translational issues should be addressed:

*Why were only seven miRNAs selected?

*Were laboratory personnel blinded to diagnosis?

*Were repeated RNA extractions performed?

*How many samples were excluded because of hemolysis?

*Were Ct values normally distributed?

*Why was miR-16 selected instead of global mean normalization?

*Were ROC curves compared using independent test datasets?

*Were stroke subtype analyses (TOAST classification) performed?

*Were biomarker levels associated with infarct volume?

*Could these biomarkers predict functional outcome?

* Reduce repetition regarding biomarker potential and expand comparison with contemporary literature like newer studies published after 2023 and meta-analysis.

*Expand discussion of limitations and clinical applicability of this study.

**Comments for Editor:**

This manuscript evaluates circulating microRNAs as early diagnostic biomarkers for AIS, HS, and TIA in a prospective case-control study of 200 participants. The work is technically sound and clinically relevant, with standardized RT-qPCR methods, predefined sample-size calculation, and appropriate internal validation only.

The novelty of the study is moderate because numerous studies have already investigated circulating miRNAs in stroke. The principal limitation is the lack of external validation, which substantially limits confidence in the reported diagnostic performance. Additional concerns include incomplete adjustment for potential confounders, reliance on healthy controls rather than clinically relevant stroke mimics, and limited discussion of real-world implementation. These issues reduce the immediate translational impact of the findings.
