# Supplementary material for: Diagnostic performance of circulating microRNAs for the early diagnosis of different types of acute cerebrovascular disease
Source: PLoS One. 2026 Oct 5;21(10):e0359648. doi: 10.1371/journal.pone.0359648 (PMC13637907; doi:10.1371/journal.pone.0359648)
Supplement: Supplementary file 4 [file pone.0359648.s005.docx]

PONE-D-26-35421

Diagnostic Performance of Circulating MicroRNAs for the Early Diagnosis of Different Types of Acute Cerebrovascular Disease

PLOS One

1. **Please ensure that your manuscript meets PLOS ONE's style requirements, including those for file naming. The PLOS ONE style templates can be found at**

<https://journals.plos.org/plosone/s/file?id=wjVg/PLOSOne_formatting_sample_main_body.pdf> and

<https://journals.plos.org/plosone/s/file?id=ba62/PLOSOne_formatting_sample_title_authors_affiliations.pdf>

We have revised the manuscript to ensure compliance with the PLOS ONE style and formatting requirements, including the formatting of the main text, title page, author names and affiliations, references, and file naming, in accordance with the provided PLOS ONE formatting guidelines.

2. **Please note that PLOS One has specific guidelines on code sharing for submissions in which author-generated code underpins the findings in the manuscript. In these cases, all author-generated code must be made available without restrictions upon publication of the work. Please review our guidelines at** [**https://journals.plos.org/plosone/s/materials-and-software-sharing#loc-sharing-code**](https://journals.plos.org/plosone/s/materials-and-software-sharing#loc-sharing-code) **and ensure that your code is shared in a way that follows best practice and facilitates reproducibility and reuse**.

In accordance with the PLOS ONE code-sharing guidelines, we have made the author-generated R code underlying the statistical analyses and figure generation publicly available without restrictions in a GitHub repository. The repository includes the analysis scripts, figure-generation code, software dependencies, and session information to facilitate reproducibility and reuse. The repository is available at: <https://github.com/AlinaKoshelyuk/circulating-miRNA-stroke-analysis>. The corresponding link has also been added to the Data Availability Statement in the revised manuscript.

**3. We note that the grant information you provided in the ‘Funding Information’ and ‘Financial Disclosure’ sections do not match.**

**When you resubmit, please ensure that you provide the correct grant numbers for the awards you received for your study in the ‘Funding Information’ section.**

**4. Thank you for stating the following financial disclosure:**

**“The authors declare that financial support was received for the research and publication of this article. This work was supported by the Ministry of Science and Higher Education of the Republic of Kazakhstan under Grant AP23490807 (Contract No. 331/GF 24–26, dated October 1, 2024); Karaganda Medical University.”**

**Please state what role the funders took in the study.  If the funders had no role, please state: "The funders had no role in study design, data collection and analysis, decision to publish, or preparation of the manuscript."**

**If this statement is not correct you must amend it as needed.**

**Please include this amended Role of Funder statement in your cover letter; we will change the online submission form on your behalf.**

**5. Thank you for stating the following financial disclosure:**

**“The authors declare that financial support was received for the research and publication of this article. This work was supported by the Ministry of Science and Higher Education of the Republic of Kazakhstan under Grant AP23490807 (Contract No. 331/GF 24–26, dated October 1, 2024); Karaganda Medical University.”**

**We note that one or more of the authors is affiliated with the funding organization, indicating the funder may have had some role in the design, data collection, analysis or preparation of your manuscript for publication; in other words, the funder played an indirect role through the participation of the co-authors. If the funding organization did not play a role in the study design, data collection and analysis, decision to publish, or preparation of the manuscript and only provided financial support in the form of authors' salaries and/or research materials, please do the following:**

**1. Review your statements relating to the author contributions, and ensure you have specifically and accurately indicated the role(s) that these authors had in your study. These amendments should be made in the online form.**

**2. Confirm in your cover letter that you agree with the following statement, and we will change the online submission form on your behalf:**

**“The funder provided support in the form of salaries for authors [insert relevant initials], but did not have any additional role in the study design, data collection and analysis, decision to publish, or preparation of the manuscript. The specific roles of these authors are articulated in the ‘author contributions’ section**.

The funding information has been reviewed and will be entered consistently in the Funding Information and Financial Disclosure sections upon resubmission. The correct grant number is **AP23490807** (Contract No. 331/GF 24–26, dated October 1, 2024). The Author Contributions information has also been reviewed to ensure that the specific roles of the authors affiliated with Karaganda Medical University are accurately stated.

The following Role of Funder statement has been included in the revised cover letter: “The funders had no role in study design, data collection and analysis, decision to publish, or preparation of the manuscript. The specific roles of the authors affiliated with Karaganda Medical University are articulated in the Author Contributions section.”

**6. In the online submission form you indicate that your data is not available for proprietary reasons and have provided a contact point for accessing this data. Please note that your current contact point is a co-author on this manuscript. According to our Data Policy, the contact point must not be an author on the manuscript and must be an institutional contact, ideally not an individual. Please revise your data statement to a non-author institutional point of contact, such as a data access or ethics committee, and send this to us via return email. Please also include contact information for the third party organization, and please include the full citation of where the data can be found**.

The Data Availability Statement has been revised to provide a non-author institutional point of contact for data access, as requested. The Local Bioethics Commission of Karaganda Medical University has been designated as the institutional contact, and its contact details have been provided. The revised statement has also been included in the cover letter as follows:

“The de-identified data underlying the findings of this study are not publicly available due to ethical and confidentiality restrictions related to participant data. Data are available upon reasonable request and subject to institutional approval. Data access requests should be directed to the Local Bioethics Commission of Karaganda Medical University, 40 Gogol Street, Karaganda, Kazakhstan; email: lkbnaomuk@gmail.com.”

**7. We notice that your supplementary tables are included in the manuscript file. Please remove them and upload them with the file type 'Supporting Information'. Please ensure that each Supporting Information file has a legend listed in the manuscript after the references list**.

The supplementary tables have been removed from the main manuscript and are now provided as separate Supporting Information files (S1 Table and S2 Table). Corresponding legends for both files have been added to the Supporting Information section after the References, in accordance with the journal’s guidelines.

**Comments to the Author**

**Reviewer #1:**

**This study addresses a clinically significant issue and evaluates circulating microRNAs in three core clinical groups of acute cerebrovascular disease at two early time points. However, the current case-control design, the absence of a true clinical stroke-mimic group, significant comorbidity imbalances between groups, normalization with a single hemolysis-sensitive reference microRNA, extensive multiple testing burden, and feature selection, cutoff determination, and ROC performance assessment on the same dataset all carry a high risk of optimism in diagnostic accuracy estimates. Furthermore, internal validation was applied to only a selected model, and optimism-corrected performance measures were not numerically reported. Therefore, the study, in its current form, does not support strong diagnostic biomarker claims for clinical application. The authors need to significantly restructure the analyses, expand the reporting, and interpret the results more cautiously within the framework of an exploratory case-control study.
1. The authors state that the control group was matched for age, gender, and cardiovascular risk factors. However, according to Table 1, all members of the control group had hypertension. Furthermore, the control group consisted of individuals "without signs of acute cerebrovascular disease." It is not explained from which clinic this group was selected, the reason for their hospital admission, whether they were asymptomatic community controls or stroke-mimic patients with neurological symptoms, how they were ruled out by CT/MRI, or whether they included clinical differential diagnoses such as headache, seizures, hypoglycemia, vertigo, migraine, or peripheral vestibular disease. A true stroke biomarker must differentiate stroke-mimic cases presenting with acute neurological symptoms from healthy or stable hypertensive controls. Therefore, the current case-control design carries a significant risk of spectrum bias and diagnostic accuracy inflation. Additionally, the groups differed significantly in terms of atrial fibrillation, coronary artery disease, heart failure, previous stroke, leukocytes, glucose, and cholesterol. For example, chronic heart failure was present in 88% of the AIS group, 40% of the control group, and 24% of the TIA group. Many of these variables can independently affect circulating microRNA levels.**

The description of the control group has been revised and substantially expanded in the Methods. The control group consisted of ambulatory patients with arterial hypertension recruited from the City Center for Primary Health Care. They had no acute neurological symptoms or focal neurological deficits at enrollment and no history of stroke or transient ischemic attack. CT or MRI was not routinely performed in controls because there was no clinical suspicion of acute cerebrovascular disease. The previous statement that controls were matched for cardiovascular risk factors has been corrected to specify that controls were matched to the patient groups for age, sex, and the presence of hypertension. The phrase “without signs of acute cerebrovascular disease” has also been removed to avoid an insufficiently precise characterization of the control population.

We have further clarified that these participants represent stable hypertensive controls rather than a clinical stroke-mimic population. Consequently, the present case-control design does not establish the ability of the investigated miRNAs to differentiate acute cerebrovascular disease from other conditions presenting with acute stroke-like symptoms. The resulting potential for spectrum bias and overestimation of diagnostic performance has been explicitly acknowledged in the revised Discussion. We also clarify that the reported diagnostic performance estimates and cut-off values should be considered exploratory and require prospective validation in clinically representative populations, including patients with relevant stroke-mimicking conditions.

In response to the concerns regarding between-group differences in cardiovascular comorbidities and laboratory parameters, all variables highlighted by the reviewer were rechecked against the individual source medical records. This verification identified an error in the previously reported prevalence of chronic heart failure in the AIS group. The original value of 44/50 (88.0%) was incorrect; the verified value is 18/50 (36.0%). Chronic heart failure data for the other study groups were also verified and confirmed to be correct. After correction, the between-group analysis was repeated, and the overall difference in chronic heart failure prevalence was no longer statistically significant (P = 0.092). Table 1 and the corresponding Results text have been revised accordingly. The remaining clinical and laboratory variables highlighted in the comment were likewise rechecked against the source data.

To further clarify the observed between-group differences, post hoc pairwise comparisons with adjustment for multiple testing were performed where appropriate. These analyses showed that some significant overall differences in Table 1 did not translate into significant differences between all individual groups. In particular, because a history of previous stroke was an exclusion criterion for controls, this variable was additionally compared among the three clinical groups only; no significant difference was observed (P = 0.284). The corresponding Results text has been revised to distinguish overall group effects from adjusted pairwise comparisons.

The multivariable diagnostic analysis was also substantially revised in response to the concern that the original adjustment for age, sex, and blood glucose alone did not adequately address potential confounding. Covariates were selected based on their established clinical relevance to cerebrovascular and cardiovascular disease and their potential association with circulating miRNA profiles, rather than solely on statistical significance in univariable comparisons. The revised clinical model therefore included age, sex, atrial fibrillation, blood glucose, and total cholesterol. Multicollinearity was assessed before model fitting, with no evidence of substantial multicollinearity (VIF < 5 for all variables). To ensure a valid direct comparison, the clinical model, let-7e-5p alone, and the combined model were evaluated in the same complete-case subset (n = 78; 39 AIS and 39 controls).

In the revised analysis, the clinical model yielded an AUC of 0.832 (95% CI, 0.742–0.923), whereas let-7e-5p alone yielded an AUC of 0.855 (95% CI, 0.767–0.942). Addition of let-7e-5p to the clinical model increased the AUC to 0.982 (95% CI, 0.954–1.000). The combined model significantly outperformed the clinical model (DeLong P < 0.001) and let-7e-5p alone (P = 0.002), whereas the clinical model and let-7e-5p alone did not differ significantly (P = 0.750). The incremental contribution of let-7e-5p was also supported by a likelihood ratio test comparing the nested clinical and combined models (χ² = 50.98, df = 1, P < 0.001). These analyses therefore indicate that the association between let-7e-5p and AIS discrimination was retained after accounting for the selected clinical covariates and that the biomarker provided incremental diagnostic information beyond the clinical model.

Given the high apparent discrimination of the combined model and the limited complete-case sample size, we additionally performed internal validation using 1,000 bootstrap resamples with optimism correction. The optimism-corrected AUC was 0.789 for the clinical model and 0.957 for the combined model, compared with apparent AUCs of 0.832 and 0.982, respectively. These results have been incorporated into the revised Results and Discussion, while the need for external validation in larger, clinically representative prospective cohorts has been emphasized. Figure 4 has also been revised to present the clinical model, let-7e-5p alone, and the combined model based on the same complete-case population.

**2. The authors used only age, sex, and blood glucose in the multivariate model. However, the main variables showing significant differences between groups in Table 1 are: atrial fibrillation, coronary artery disease, chronic heart failure, previous stroke, leukocyte count, total cholesterol, anticoagulant use, neurological severity/NIHSS. The fact that chronic heart failure was present in 88% of the AIS group is particularly noteworthy and its accuracy should be re-checked. If this rate is accurate, circulating miRNA profiles may reflect the burden of cardiovascular comorbidities rather than acute stroke. Including only glucose in the model is insufficient, and no predetermined clinical/statistical justification for variable selection has been provided.**

The clinical and laboratory variables highlighted by the reviewer were systematically re-evaluated before reconstruction of the multivariable diagnostic model. In addition to the overall comparisons presented in Table 1, adjusted post hoc pairwise comparisons were performed where appropriate to determine which differences were specifically present between the groups relevant to the diagnostic analysis.

The unexpectedly high prevalence of chronic heart failure in the AIS group was rechecked against the individual source medical records. This verification identified an error in the originally reported value of 44/50 (88.0%); the correct prevalence was 18/50 (36.0%). The corresponding data for the other study groups were also verified against the source records and confirmed to be correct. After correction and repetition of the between-group analysis, chronic heart failure no longer differed significantly across the study groups (P = 0.092). Table 1 and the corresponding Results text have been revised accordingly. The other clinical and laboratory variables highlighted by the reviewer were likewise rechecked against the source data.

We also reconsidered each of the variables identified by the reviewer with respect to its suitability for adjustment in the AIS-versus-control diagnostic model. **NIHSS** was not included because it quantifies the severity of an acute neurological deficit and is not applicable to the control population; incorporating it into an AIS-versus-control model would therefore introduce information intrinsically related to acute case status rather than an appropriate baseline covariate. **Previous stroke** was also not included because a history of stroke was an exclusion criterion for controls and was therefore structurally related to group definition. To clarify the overall difference reported in Table 1, this variable was additionally compared among the three clinical groups only, with no significant difference observed (P = 0.284). **Chronic heart failure** was not retained after verification of the source data, as the corrected analysis showed no significant overall between-group difference (P = 0.092). **Leukocyte count** was not included because the adjusted pairwise comparison did not demonstrate a significant difference between AIS and controls (Holm-adjusted P = 0.068), despite differences between controls and some of the other clinical groups. **Coronary artery disease** showed an overall difference across the four groups (P = 0.037), but this was not observed between AIS and controls in adjusted pairwise analysis; the only significant pairwise difference was between TIA and controls (adjusted P = 0.038). CAD was therefore not retained as an additional covariate. Given the limited complete-case sample, model parsimony was also considered to avoid unnecessary inclusion of multiple cardiovascular comorbidities. **Anticoagulant use** was not included in the revised multivariable model because information on pre-admission anticoagulant therapy was collected specifically for participants with documented atrial fibrillation rather than systematically for the entire study population. Accordingly, this variable represents a treatment characteristic within the AF subgroup and was therefore not considered appropriate for inclusion as an independently ascertained covariate in the AIS-versus-control model.

Accordingly, covariate selection was not based solely on statistical significance in the overall four-group comparisons. The revised clinical model included **age, sex, atrial fibrillation, blood glucose, and total cholesterol**, selected as clinically relevant potential confounders that could be assessed in both AIS and control participants. Multicollinearity was evaluated before model fitting using variance inflation factors and tolerance, with no evidence of substantial multicollinearity (VIF < 5 for all variables).

The diagnostic analyses were then repeated in a common complete-case subset (n = 78; 39 AIS and 39 controls), ensuring that the clinical model, let-7e-5p alone, and the combined model were compared in the same participants. The clinical model yielded an AUC of 0.832 (95% CI, 0.742–0.923), whereas let-7e-5p alone yielded an AUC of 0.855 (95% CI, 0.767–0.942). Addition of let-7e-5p increased the AUC to 0.982 (95% CI, 0.954–1.000). The combined model significantly outperformed the clinical model (DeLong P < 0.001) and let-7e-5p alone (P = 0.002), whereas no significant difference was observed between the clinical model and let-7e-5p alone (P = 0.750). The incremental contribution of let-7e-5p was further supported by the likelihood ratio test comparing the nested clinical and combined models (χ² = 50.98, df = 1, P < 0.001).

Given the limited complete-case sample and the high apparent discrimination of the combined model, **both the clinical and combined logistic regression models were subjected to internal validation using 1,000 bootstrap resamples with optimism correction**. The apparent and optimism-corrected AUCs were 0.832 and 0.789, respectively, for the clinical model and 0.982 and 0.957, respectively, for the combined model. The revised Methods, Results, Discussion, and Fig 4 have been updated to reflect the revised covariate-selection strategy, model comparisons, and internal validation.

**3. Only patients with a referral NIHSS score of 3–23 were included in the study. This selection excludes minor strokes with an NIHSS score <3 and severe strokes with an NIHSS score >23. However, diagnostic uncertainty is higher, and the need for biomarkers is more pronounced, particularly in TIA and minor stroke. Such a limitation narrows the clinical spectrum and prevents the transfer of the obtained cutoff/AUC values to the general stroke population. The fact that the median NIHSS score in the TIA group was 3 also needs to be explained. Were the symptoms present at the time of sampling or evaluation in TIA? Were the patients truly classified as having TIA or as having imaging-negative minor stroke?**

We have clarified the timing and interpretation of NIHSS assessment in the TIA group. The NIHSS score reported in the study was assessed at hospital admission, when focal neurological symptoms were still present. In patients ultimately classified as having TIA, neurological symptoms completely resolved within 24 h, with an NIHSS score of 0 at 24 h. All patients underwent CT and/or MRI, and no acute ischemic lesion was identified in the TIA group. The final diagnosis of TIA was established by a neurologist based on the transient clinical presentation, complete resolution of neurological symptoms within 24 h, and the absence of acute ischemic lesions on neuroimaging. These details have been added to the Methods section.

We have also acknowledged that restricting enrollment to patients with admission NIHSS scores of 3–23 narrowed the clinical spectrum of the study population and limits the generalizability of the reported AUC and cut-off values, particularly to patients with minor stroke. This limitation has been added to the Discussion.

**4. Normalization was performed solely with miR-16-5p. The rationale was based on two previous studies, which showed no difference in Ct between the groups. However, the absence of statistically significant differences between groups does not prove that a reference gene is biologically and technically stable. The main issues are:
a. miR-16-5p may be affected by hemolysis.
b. Only one reference was used.
c. Stability analyses such as NormFinder, geNorm, or BestKeeper were not performed.
d. Comparison of candidate references was not presented.
e. Control Ct values were reused in two time tables because only a single sample was taken from the control group.**

miR-16-5p was selected a priori as the endogenous reference based on previous studies and was measured consistently across all samples. In the present dataset, its raw Ct values showed limited between-group variation and did not differ significantly among the study groups at either sampling time point (P = 0.72 at 3–4.5 h and P = 0.81 at 12 h; S2 Table).

Potential hemolysis-related effects were addressed by assessing hemolysis in all plasma samples using cell-free hemoglobin measurements, as described in the Methods, and excluding hemolyzed samples from further analysis.

As miR-16-5p was the only endogenous reference miRNA measured across all samples, comparison with alternative reference miRNAs and retrospective application of stability algorithms such as geNorm, NormFinder, or BestKeeper were not possible. This limitation has been explicitly stated in the revised Discussion.

Control participants underwent a single blood collection, whereas patients were sampled at 3–4.5 h and 12 h after symptom onset. Accordingly, the same control measurements were used as the reference for cross-sectional comparisons at both patient sampling time points. This has been clarified in the revised Methods, and comparisons with controls at the two time points were not interpreted as independent replication.

**5. Two samples were taken from the patient groups at 3–4.5 and 12 hours, while only one sample was taken from the control group. The repeated use of the same control values in analyses at two different time points should be carefully considered in terms of independence of comparisons, multiple test burden, and interpretation of time effects.**

The same control measurements were used as the reference at both patient sampling time points because blood sampling was performed once in the control group. This aspect of the analytical design has been clarified in the revised manuscript. Comparisons of the 3–4.5-h and 12-h patient samples with the control group were considered separate cross-sectional comparisons and were not treated as independent replications. Accordingly, differences in statistical significance relative to controls between the two time points were not interpreted as evidence of temporal changes in miRNA expression. Temporal changes were evaluated separately within each clinical group using paired measurements obtained from the same patients at 3–4.5 h and 12 h and analyzed with the Wilcoxon signed-rank test. To make this distinction explicit, the Statistical Analysis section now states that “within-group changes in miRNA expression between 3–4.5 h and 12 h were assessed using the Wilcoxon signed-rank test, whereas control measurements were used only for cross-sectional comparisons at each patient sampling time point.”

The multiple-testing strategy was also further examined in light of this comment. For the primary pairwise analyses, P values were adjusted using the Holm–Bonferroni procedure across the six group comparisons within each miRNA and time point. With seven miRNAs evaluated at two time points, this resulted in 84 pairwise tests. As an additional sensitivity analysis, the Benjamini–Hochberg false discovery rate (FDR) procedure was applied across all 84 tests. Of these, 43 had unadjusted P values < 0.05, 36 remained significant after Holm–Bonferroni correction, and 42 met the FDR-adjusted significance threshold. Importantly, all 36 comparisons that remained significant after Holm–Bonferroni correction also retained statistical significance after the global FDR adjustment. Thus, the findings retained under the primary correction procedure were robust to broader control of the multiple-testing burden. The Statistical Analysis and Results sections have been revised accordingly.

**6. The application of the 2^-ΔΔCt approach and the presentation of fold-change are not sufficiently explained. Some very large reductions are reported in the tables; for example, for let-7e-5p, FC drops to the range of 0.007–0.061. This represents a reduction of approximately 94–99% and has quite strong biological effects. However, it is unclear whether ΔΔCt is calculated from group medians or individual values, whether the statistical test is performed on ΔCt or FC, the status of distributions and outliers, and whether the amplification efficiencies are approximately equal.**

For each sample, ΔCt was calculated as Ct(target miRNA) − Ct(miR-16-5p). ΔΔCt was then calculated by subtracting the median ΔCt of the control group from the individual ΔCt value. Relative expression was expressed as fold change (FC) using the 2^−ΔΔCt method. Thus, ΔΔCt values were calculated at the individual-sample level rather than from group-level median ΔCt values. The low FC values observed for let-7e-5p were derived from individual ΔΔCt values and were used to describe relative expression; all inferential statistical analyses were performed using individual ΔCt values rather than FC values. The distribution of ΔCt values was assessed using the Shapiro–Wilk test, and no observations were excluded solely on the basis of extreme ΔCt values. Amplification efficiencies were evaluated using standard curves generated from serial dilutions. The standard curves demonstrated satisfactory linearity across the tested dilution range, and the amplification efficiencies of the target miRNA assays were comparable to that of the endogenous reference miR-16-5p. These findings supported the use of the 2^−ΔΔCt method for relative quantification.

**7. Most correlations are weak or moderate, and no multiple comparison correction was applied. A correlation between miR-107 and leukocytes with a P<0.05 at two time points was defined as "reproducible". However, the two time points are not independent in the same individuals. Therefore, two significant results cannot be considered independent replication.**

The correlation analysis was revised to account for multiple comparisons and for the non-independence of repeated measurements. Benjamini–Hochberg FDR correction was applied to the exploratory correlation analyses within each clinical group and sampling time point. After FDR adjustment, none of the nominal associations remained statistically significant (all adjusted P > 0.05). The Results section has been revised accordingly, and the correlations are no longer presented as statistically significant findings after correction for multiple testing.

The interpretation of the association between miR-107 expression and leukocyte count in the AIS group was also revised. Although nominal inverse associations were observed at both 3–4.5 h (r = −0.343; P = 0.041) and 12 h (r = −0.396; P = 0.013), these measurements were obtained from the same individuals and therefore cannot be regarded as independent replication. Moreover, neither association remained statistically significant after FDR correction. The term “reproducible” has consequently been removed from the manuscript, and the corresponding figure has also been removed to avoid overemphasizing an exploratory association that did not withstand correction for multiple testing. The Statistical Analysis section has been updated to specify the use of Benjamini–Hochberg FDR correction for the exploratory correlation analysis.

**8. This study only examines circulating expression and diagnostic performance. However, the discussion suggests that these miRNAs could be therapeutic targets. This study lacks: target gene analysis, functional experiment, cellular validation, protein level, and causality analysis. Therefore, the therapeutic target claim is speculative and deviates from the diagnostic focus of the article.**

No target gene analysis, functional experiments, cellular validation, protein-level assessment, or causal analyses were performed. Therefore, statements suggesting that the investigated miRNAs may represent therapeutic targets were considered beyond the scope of the present study and have been removed from the revised Discussion. The interpretation has been refocused on their potential role as circulating diagnostic biomarkers, with appropriate emphasis on the exploratory nature of the findings and the need for external validation.

**Reviewer #2:**

**1st, what is the mean time from onset to blood sample drawing for miRNA estimation?**

The onset-to-sampling times for both blood collections are provided below as mean ± SD, as requested, together with the corresponding median (IQR) values. In the revised manuscript, these data are reported as median (IQR), consistent with the presentation of continuous variables and the statistical approach used for between-group comparisons. Onset-to-sampling times did not differ significantly among the AIS, HS, and TIA groups at either the first (P = 0.126) or second (P = 0.695) sampling time point. These data have been added to the Results section.

| **Sampling point** | **Group** | **Mean ± SD, h** | **Median (IQR), h** |
| --- | --- | --- | --- |
| First sampling | AIS | 3.80 ± 0.39 | 3.8 (3.4–4.2) |
|  | HS | 3.71 ± 0.40 | 3.7 (3.3–4.1) |
|  | TIA | 3.87 ± 0.37 | 3.9 (3.5–4.2) |
| Second sampling | AIS | 12.03 ± 0.14 | 12.0 (11.9–12.2) |
|  | HS | 12.00 ± 0.11 | 12.0 (11.9–12.1) |
|  | TIA | 12.03 ± 0.14 | 12.0 (11.9–12.2) |

**2nd What would be the time to get a quatifiable valuae to miRNA (Estimation time)?
As we know, stroke, especially the ischemic one, may be significantly reverse by adequate treatment by either medical or mechanical thrombectomy within the strict time window.
Will these biomarker estimates produce much faster results?
As far as stroke diagnosis is concern it is clinical. the Imaging modalities will only help to clasify in different types and subtypes of stroke**.

The miRNA measurements in the present study were performed using a laboratory-based RT-qPCR workflow involving plasma processing, RNA extraction, cDNA preparation, qPCR amplification, and subsequent data analysis, with a total analytical turnaround time of approximately 5–6 h. Therefore, the workflow used in this study was intended to evaluate the diagnostic potential of circulating miRNAs rather than to provide results for immediate clinical decision-making within the time-sensitive reperfusion window of acute stroke. At present, this laboratory-based approach is not sufficiently rapid to guide decisions regarding intravenous thrombolysis or mechanical thrombectomy. Circulating miRNA biomarkers should therefore be considered potentially complementary to, rather than a replacement for, established clinical assessment and neuroimaging in acute stroke diagnosis and differentiation. Further development of rapid, automated, and point-of-care nucleic acid detection platforms may reduce the turnaround time for miRNA quantification and facilitate their future evaluation in acute stroke diagnostics. This consideration has been added to the revised Discussion.

**Reviewer #3:**

***Why were only seven miRNAs selected?**

The seven miRNAs were selected a priori based on the findings of our previously published systematic review and the available literature, with a focus on circulating miRNAs previously reported to show differential expression and potential diagnostic relevance in acute cerebrovascular disease. The study was designed as a targeted evaluation of a predefined candidate miRNA panel rather than an exploratory high-throughput screening study. The rationale for selecting these seven miRNAs has been further clarified in the Introduction and Methods.

***Were laboratory personnel blinded to diagnosis?**

Laboratory personnel were blinded to the participants’ clinical diagnoses and group assignments. All samples were assigned coded identifiers before laboratory analysis, and RNA extraction and RT-qPCR were performed using coded samples. This information has been added to the Methods section of the revised manuscript.

***Were repeated RNA extractions performed**?

A single RNA extraction was performed for each plasma sample using the standardized MagMAX mirVana Total RNA Isolation Kit protocol. Independent replicate RNA extractions from the same plasma sample were not routinely performed.

***How many samples were excluded because of hemolysis?**

Three participants were excluded from the final analysis because hemolysis was detected in their first plasma samples. Repeat blood sampling was not performed because the predefined 3–4.5-h sampling window had elapsed. Recruitment subsequently continued until the predefined sample size was reached. This information has now been added to the Results section of the revised manuscript.

***Were Ct values normally distributed?**

Ct values were assessed for normality using the Shapiro–Wilk test and were not consistently normally distributed across the investigated miRNAs, study groups, and sampling time points. Therefore, miRNA expression data were summarized using medians and interquartile ranges (IQR), and nonparametric statistical methods were used for the corresponding analyses. The assessment of distribution and the use of median (IQR) for continuous variables are specified in the Statistical Analysis section.

***Why was miR-16 selected instead of global mean normalization?**

miR-16-5p was selected as the endogenous reference miRNA based on previous studies supporting its use for normalization of circulating miRNA expression. Global mean normalization was not applied because the present study used a targeted panel of only seven candidate miRNAs rather than a high-throughput miRNA profiling approach. These candidate miRNAs were selected because of their previously reported differential expression in acute cerebrovascular disease, making their global mean unsuitable as an independent normalization reference. In the present dataset, raw miR-16-5p Ct values showed limited variation and no significant differences among the study groups at either sampling time point (Supplementary Table S2). However, because miR-16-5p was the only endogenous reference miRNA measured, comparative reference-miRNA stability analyses could not be performed, and this has been acknowledged as a limitation of the study.

***Were ROC curves compared using independent test datasets?**

ROC curves were generated and compared within the same study cohort; an independent test dataset was not available. For ROC curves based on measurements obtained from the same participants, comparisons were performed using DeLong’s test for correlated ROC curves. The absence of an independent external validation cohort is acknowledged as a limitation of the study.

***Were stroke subtype analyses (TOAST classification) performed?**

An exploratory analysis according to TOAST classification was performed in the revised manuscript. Among the 50 patients with AIS, 30 (60.0%) were classified as having large-artery atherosclerosis, 13 (26.0%) as cardioembolism, and 7 (14.0%) as stroke of undetermined etiology; no cases of small-vessel occlusion or stroke of other determined etiology were identified. Expression levels of all seven investigated miRNAs were compared among the three represented TOAST subtypes separately at 3–4.5 h and 12 h after symptom onset using the Kruskal–Wallis test. No significant differences in miRNA expression were identified among the TOAST subtypes at either sampling time point (all P > 0.05). The corresponding findings have been added to the Results section of the revised manuscript.

***Were biomarker levels associated with infarct volume?**

Biomarker levels were not analyzed in relation to infarct volume because quantitative infarct volume measurements were not systematically available in the medical records for the study cohort. Performing this analysis only in the subset of patients with available volumetric imaging data could introduce selection bias and would not provide a reliable assessment of the association between miRNA expression and infarct volume. We have acknowledged the lack of systematic infarct volume assessment as a limitation of the study.

***Could these biomarkers predict functional outcome?**

We performed an additional exploratory analysis of functional outcome at hospital discharge separately in patients with AIS and HS. Associations between miRNA expression and the full modified Rankin Scale (mRS) score (0–6) were evaluated using Spearman's rank correlation at both sampling time points. In addition, miRNA expression was compared between patients with favorable (mRS 0–2) and unfavorable (mRS 3–6) outcomes using the Mann–Whitney U test. Multiple testing was addressed using the Benjamini–Hochberg false discovery rate (FDR) procedure.

No associations remained statistically significant after FDR adjustment. The strongest nominal association was observed in the HS group at 12 h, where miR-143-3p expression was positively correlated with the mRS score at discharge (r = 0.435, unadjusted P = 0.008); however, this association did not retain statistical significance after FDR correction (adjusted P = 0.057). At the same time point, differences between favorable and unfavorable outcome groups were observed before correction for let-7e-5p (unadjusted P = 0.043) and miR-143-3p (unadjusted P = 0.027), but neither remained significant after FDR adjustment (adjusted P = 0.149 for both). No significant associations with functional outcome were identified in the AIS group.

These analyses have been incorporated into the revised manuscript. Overall, the present findings do not provide statistically robust evidence of an association between early miRNA expression and functional outcome at discharge. Because functional outcome was available only at hospital discharge and not at a standardized longer-term follow-up such as 90 days, the prognostic value of the investigated miRNAs cannot be established from the present study and should be evaluated in appropriately designed longitudinal cohorts.

***Reduce repetition regarding biomarker potential and expand comparison with contemporary literature like newer studies published after 2023 and meta-analysis**.

We have revised the Discussion to reduce repetitive statements regarding the biomarker potential of circulating miRNAs and to provide a more balanced interpretation of our findings. We have also expanded the comparison with contemporary literature by incorporating recent studies published after 2023, including systematic reviews and meta-analyses, and discussing our findings in the context of these newer data.

***Expand discussion of limitations and clinical applicability of this study**.

We have expanded the Discussion to provide a more detailed consideration of the study limitations and clinical applicability. In particular, we now address the case-control design, the use of stable controls rather than patients with stroke-mimicking conditions, potential effects of cardiovascular comorbidities, sample size, normalization strategy, reuse of the control measurements across sampling time points, and the lack of independent external validation. We have also clarified that the current RT-qPCR-based approach is intended as a complementary research tool rather than a replacement for established clinical and neuroimaging assessment, and that further prospective validation in larger, independent cohorts, including patients with stroke mimics, is required before clinical implementation.

**To ensure your figures meet our technical requirements, please review our figure guideline**s: <https://journals.plos.org/plosone/s/figures>

We carefully reviewed all figures in accordance with the PLOS ONE figure guidelines and revised them as needed to ensure compliance with the journal’s technical and formatting requirements.
