# Supplementary material for: Diagnostic performance of circulating microRNAs for the early diagnosis of different types of acute cerebrovascular disease
Source: PLoS One. 2026 Oct 5;21(10):e0359648. doi: 10.1371/journal.pone.0359648 (PMC13637907; doi:10.1371/journal.pone.0359648)
Supplement: Peer review history

# Peer review: Decision Letter 0

**DOI:** 10.1371/journal.pone.0359648.r001

**Authors:** Francesco Sessa

9 Aug 2026

Dear Dr. Koshelyuk,

Thank you for submitting your manuscript to PLOS ONE. After careful consideration, we feel that it has merit but does not fully meet PLOS ONE’s publication criteria as it currently stands. Therefore, we invite you to submit a revised version of the manuscript that addresses the points raised during the review process.

Please submit your revised manuscript by Oct 08 2026 11:59PM. If you will need more time than this to complete your revisions, please reply to this message or contact the journal office at plosone@plos.org. When you're ready to submit your revision, log on to https://www.editorialmanager.com/pone/ and select the 'Submissions Needing Revision' folder to locate your manuscript file.

- A letter that responds to each point raised by the academic editor and reviewer(s). You should upload this letter as a separate file labeled 'Response to Reviewers'.
- A marked-up copy of your manuscript that highlights changes made to the original version. You should upload this as a separate file labeled 'Revised Manuscript with Track Changes'.
- An unmarked version of your revised paper without tracked changes. You should upload this as a separate file labeled 'Manuscript'.

If you would like to make changes to your financial disclosure, please include your updated statement in your cover letter. Guidelines for resubmitting your figure files are available below the reviewer comments at the end of this letter.

If applicable, we recommend that you deposit your laboratory protocols in protocols.io to enhance the reproducibility of your results. Protocols.io assigns your protocol its own identifier (DOI) so that it can be cited independently in the future. For instructions see: https://journals.plos.org/plosone/s/submission-guidelines#loc-laboratory-protocols. Additionally, PLOS ONE offers an option for publishing peer-reviewed Lab Protocol articles, which describe protocols hosted on protocols.io. Read more information on sharing protocols at https://plos.org/protocols?utm_medium=editorial-email&utm_source=authorletters&utm_campaign=protocols.

As the corresponding author, your ORCID iD is verified in the submission system and will appear in the published article. PLOS supports the use of ORCID, and we encourage all coauthors to register for an ORCID iD and use it as well. Please encourage your coauthors to verify their ORCID iD within the submission system before final acceptance, as unverified ORCID iDs will not appear in the published article. *Only*  the individual author can complete the verification step; PLOS staff *cannot*  verify ORCID iDs on behalf of authors.

We look forward to receiving your revised manuscript.

Kind regards,

Francesco Sessa, Ph.D., MS

Academic Editor

PLOS One

Journal Requirements:

When submitting your revision, we need you to address these additional requirements.

1. Please ensure that your manuscript meets PLOS ONE's style requirements, including those for file naming. The PLOS ONE style templates can be found at

https://journals.plos.org/plosone/s/file?id=wjVg/PLOSOne_formatting_sample_main_body.pdf and

https://journals.plos.org/plosone/s/file?id=ba62/PLOSOne_formatting_sample_title_authors_affiliations.pdf

2. Please note that PLOS One has specific guidelines on code sharing for submissions in which author-generated code underpins the findings in the manuscript. In these cases, all author-generated code must be made available without restrictions upon publication of the work. Please review our guidelines at https://journals.plos.org/plosone/s/materials-and-software-sharing#loc-sharing-code and ensure that your code is shared in a way that follows best practice and facilitates reproducibility and reuse.

3. We note that the grant information you provided in the ‘Funding Information’ and ‘Financial Disclosure’ sections do not match.

When you resubmit, please ensure that you provide the correct grant numbers for the awards you received for your study in the ‘Funding Information’ section.

4. Thank you for stating the following financial disclosure:

“The authors declare that financial support was received for the research and publication of this article. This work was supported by the Ministry of Science and Higher Education of the Republic of Kazakhstan under Grant AP23490807 (Contract No. 331/GF 24–26, dated October 1, 2024); Karaganda Medical University.”

Please state what role the funders took in the study.  If the funders had no role, please state: "The funders had no role in study design, data collection and analysis, decision to publish, or preparation of the manuscript."

If this statement is not correct you must amend it as needed.

Please include this amended Role of Funder statement in your cover letter; we will change the online submission form on your behalf.

5. Thank you for stating the following financial disclosure:

“The authors declare that financial support was received for the research and publication of this article. This work was supported by the Ministry of Science and Higher Education of the Republic of Kazakhstan under Grant AP23490807 (Contract No. 331/GF 24–26, dated October 1, 2024); Karaganda Medical University.”

We note that one or more of the authors is affiliated with the funding organization, indicating the funder may have had some role in the design, data collection, analysis or preparation of your manuscript for publication; in other words, the funder played an indirect role through the participation of the co-authors. If the funding organization did not play a role in the study design, data collection and analysis, decision to publish, or preparation of the manuscript and only provided financial support in the form of authors' salaries and/or research materials, please do the following:

1. Review your statements relating to the author contributions, and ensure you have specifically and accurately indicated the role(s) that these authors had in your study. These amendments should be made in the online form.

2. Confirm in your cover letter that you agree with the following statement, and we will change the online submission form on your behalf:

“The funder provided support in the form of salaries for authors [insert relevant initials], but did not have any additional role in the study design, data collection and analysis, decision to publish, or preparation of the manuscript. The specific roles of these authors are articulated in the ‘author contributions’ section.

6. In the online submission form you indicate that your data is not available for proprietary reasons and have provided a contact point for accessing this data. Please note that your current contact point is a co-author on this manuscript. According to our Data Policy, the contact point must not be an author on the manuscript and must be an institutional contact, ideally not an individual. Please revise your data statement to a non-author institutional point of contact, such as a data access or ethics committee, and send this to us via return email. Please also include contact information for the third party organization, and please include the full citation of where the data can be found.

7. We notice that your supplementary tables are included in the manuscript file. Please remove them and upload them with the file type 'Supporting Information'. Please ensure that each Supporting Information file has a legend listed in the manuscript after the references list.

8. If the reviewer comments include a recommendation to cite specific previously published works, please review and evaluate these publications to determine whether they are relevant and should be cited. There is no requirement to cite these works unless the editor has indicated otherwise.

Additional Editor Comments:

Reviewers raised several important concerns that should be addressed before the manuscript can be considered for publication.

[Note: HTML markup is below. Please do not edit.]

Reviewers' comments:

Reviewer's Responses to Questions

**Comments to the Author**

1. Is the manuscript technically sound, and do the data support the conclusions?

Reviewer #1: Partly

Reviewer #2: Yes

Reviewer #3: Partly

**********

2. Has the statistical analysis been performed appropriately and rigorously?

Reviewer #1: Yes

Reviewer #2: Yes

Reviewer #3: Yes

**********

3. Have the authors made all data underlying the findings in their manuscript fully available?

The PLOS Data policy

Reviewer #1: No

Reviewer #2: Yes

Reviewer #3: Yes

**********

4. Is the manuscript presented in an intelligible fashion and written in standard English?

Reviewer #1: Yes

Reviewer #2: Yes

Reviewer #3: Yes

**********

Reviewer #1: This study addresses a clinically significant issue and evaluates circulating microRNAs in three core clinical groups of acute cerebrovascular disease at two early time points. However, the current case-control design, the absence of a true clinical stroke-mimic group, significant comorbidity imbalances between groups, normalization with a single hemolysis-sensitive reference microRNA, extensive multiple testing burden, and feature selection, cutoff determination, and ROC performance assessment on the same dataset all carry a high risk of optimism in diagnostic accuracy estimates. Furthermore, internal validation was applied to only a selected model, and optimism-corrected performance measures were not numerically reported. Therefore, the study, in its current form, does not support strong diagnostic biomarker claims for clinical application. The authors need to significantly restructure the analyses, expand the reporting, and interpret the results more cautiously within the framework of an exploratory case-control study.

1. The authors state that the control group was matched for age, gender, and cardiovascular risk factors. However, according to Table 1, all members of the control group had hypertension. Furthermore, the control group consisted of individuals "without signs of acute cerebrovascular disease." It is not explained from which clinic this group was selected, the reason for their hospital admission, whether they were asymptomatic community controls or stroke-mimic patients with neurological symptoms, how they were ruled out by CT/MRI, or whether they included clinical differential diagnoses such as headache, seizures, hypoglycemia, vertigo, migraine, or peripheral vestibular disease. A true stroke biomarker must differentiate stroke-mimic cases presenting with acute neurological symptoms from healthy or stable hypertensive controls. Therefore, the current case-control design carries a significant risk of spectrum bias and diagnostic accuracy inflation. Additionally, the groups differed significantly in terms of atrial fibrillation, coronary artery disease, heart failure, previous stroke, leukocytes, glucose, and cholesterol. For example, chronic heart failure was present in 88% of the AIS group, 40% of the control group, and 24% of the TIA group. Many of these variables can independently affect circulating microRNA levels.

2. The authors used only age, sex, and blood glucose in the multivariate model. However, the main variables showing significant differences between groups in Table 1 are: atrial fibrillation, coronary artery disease, chronic heart failure, previous stroke, leukocyte count, total cholesterol, anticoagulant use, neurological severity/NIHSS. The fact that chronic heart failure was present in 88% of the AIS group is particularly noteworthy and its accuracy should be re-checked. If this rate is accurate, circulating miRNA profiles may reflect the burden of cardiovascular comorbidities rather than acute stroke. Including only glucose in the model is insufficient, and no predetermined clinical/statistical justification for variable selection has been provided.

3. Only patients with a referral NIHSS score of 3–23 were included in the study. This selection excludes minor strokes with an NIHSS score <3 and severe strokes with an NIHSS score >23. However, diagnostic uncertainty is higher, and the need for biomarkers is more pronounced, particularly in TIA and minor stroke. Such a limitation narrows the clinical spectrum and prevents the transfer of the obtained cutoff/AUC values to the general stroke population. The fact that the median NIHSS score in the TIA group was 3 also needs to be explained. Were the symptoms present at the time of sampling or evaluation in TIA? Were the patients truly classified as having TIA or as having imaging-negative minor stroke?

4. Normalization was performed solely with miR-16-5p. The rationale was based on two previous studies, which showed no difference in Ct between the groups. However, the absence of statistically significant differences between groups does not prove that a reference gene is biologically and technically stable. The main issues are:

a. miR-16-5p may be affected by hemolysis.

b. Only one reference was used.

c. Stability analyses such as NormFinder, geNorm, or BestKeeper were not performed.

d. Comparison of candidate references was not presented.

e. Control Ct values were reused in two time tables because only a single sample was taken from the control group.

5. Two samples were taken from the patient groups at 3–4.5 and 12 hours, while only one sample was taken from the control group. The repeated use of the same control values in analyses at two different time points should be carefully considered in terms of independence of comparisons, multiple test burden, and interpretation of time effects.

6. The application of the 2^-ΔΔCt approach and the presentation of fold-change are not sufficiently explained. Some very large reductions are reported in the tables; for example, for let-7e-5p, FC drops to the range of 0.007–0.061. This represents a reduction of approximately 94–99% and has quite strong biological effects. However, it is unclear whether ΔΔCt is calculated from group medians or individual values, whether the statistical test is performed on ΔCt or FC, the status of distributions and outliers, and whether the amplification efficiencies are approximately equal.

7. Most correlations are weak or moderate, and no multiple comparison correction was applied. A correlation between miR-107 and leukocytes with a P<0.05 at two time points was defined as "reproducible". However, the two time points are not independent in the same individuals. Therefore, two significant results cannot be considered independent replication.

8. This study only examines circulating expression and diagnostic performance. However, the discussion suggests that these miRNAs could be therapeutic targets. This study lacks: target gene analysis, functional experiment, cellular validation, protein level, and causality analysis. Therefore, the therapeutic target claim is speculative and deviates from the diagnostic focus of the article.

Reviewer #2: Nice work by the authors

There are a few concerns regarding this manuscript. 1st, what is the mean time from onset to blood sample drawing for miRNA estimation?

2nd What would be the time to get a quatifiable valuae to miRNA (Estimation time)?

As we know, stroke, especially the ischemic one, may be significantly reverse by adequate treatment by either medical or mechanical thrombectomy within the strict time window.

Will these biomarker estimates produce much faster results?

As far as stroke diagnosis is concern it is clinical. the Imaging modalities will only help to clasify in different types and subtypes of stroke

Reviewer #3: Although the topic is timely and clinically relevant, several methodological, statistical, and translational issues should be addressed:

*Why were only seven miRNAs selected?

*Were laboratory personnel blinded to diagnosis?

*Were repeated RNA extractions performed?

*How many samples were excluded because of hemolysis?

*Were Ct values normally distributed?

*Why was miR-16 selected instead of global mean normalization?

*Were ROC curves compared using independent test datasets?

*Were stroke subtype analyses (TOAST classification) performed?

*Were biomarker levels associated with infarct volume?

*Could these biomarkers predict functional outcome?

*Reduce repetition regarding biomarker potential and expand comparison with contemporary literature like newer studies published after 2023 and meta-analysis.

*Expand discussion of limitations and clinical applicability of this study.

**********

what does this mean?). If published, this will include your full peer review and any attached files.

If you choose “no”, your identity will remain anonymous but your review may still be made public.

**Do you want your identity to be public for this peer review?**  For information about this choice, including consent withdrawal, please see our Privacy Policy

Reviewer #1: **Yes:**  Ebru DERİCİ EKER

Reviewer #2: **Yes:**  Anand Kumar, MD, DM

Reviewer #3: **Yes:**  Rameshwar Nath Chaurasia

**********

[NOTE: If reviewer comments were submitted as an attachment file, they will be attached to this email and accessible via the submission site. Please log into your account, locate the manuscript record, and check for the action link "View Attachments". If this link does not appear, there are no attachment files.]

To ensure your figures meet our technical requirements, please review our figure guidelines: https://journals.plos.org/plosone/s/figures

You may also use PLOS’s free figure tool, NAAS, to help you prepare publication quality figures: https://journals.plos.org/plosone/s/figures#loc-tools-for-figure-preparation.

NAAS will assess whether your figures meet our technical requirements by comparing each figure against our figure specifications.

Attachment Submitted filename: Reviewer comments for PLOS ONE.docx

# Author comment: Author response to Decision Letter 1

**DOI:** 10.1371/journal.pone.0359648.r002

8 Sep 2026

Thank you for reviewing our revised submission. We have addressed the requested funding-related issue. The Author Contributions in the online submission form have been reviewed to ensure that the specific roles of the relevant authors are accurately indicated. We have also updated the Cover Letter to confirm our agreement with the requested Role of Funder statement. No other changes have been made to the revised manuscript in response to this technical request.

Attachment Submitted filename: Response to Reviewers.docx

# Peer review: Decision Letter 1

**DOI:** 10.1371/journal.pone.0359648.r003

**Authors:** Francesco Sessa

16 Sep 2026

Diagnostic performance of circulating microRNAs for the early diagnosis of different types of acute cerebrovascular disease

PONE-D-26-35421R1

Dear Dr. Koshelyuk,

We’re pleased to inform you that your manuscript has been judged scientifically suitable for publication and will be formally accepted for publication once it meets all outstanding technical requirements.

Within one week, you’ll receive an e-mail detailing the required amendments. When these have been addressed, you’ll receive a formal acceptance letter and your manuscript will be scheduled for publication.

An invoice will be generated when your article is formally accepted. Please note, if your institution has a publishing partnership with PLOS and your article meets the relevant criteria, all or part of your publication costs will be covered. Please make sure your user information is up-to-date by logging into Editorial Manager at Editorial Manager® and clicking the ‘Update My Information' link at the top of the page. For questions related to billing, please contact billing support.

If your institution or institutions have a press office, please notify them about your upcoming paper to help maximize its impact. If they’ll be preparing press materials, please inform our press team as soon as possible -- no later than 48 hours after receiving the formal acceptance. Your manuscript will remain under strict press embargo until 2 pm Eastern Time on the date of publication. For more information, please contact onepress@plos.org.

Kind regards,

Francesco Sessa, Ph.D., MS

Academic Editor

PLOS One

Additional Editor Comments (optional):

Following the reviewers' suggestions, the authors improved their manuscript.

Reviewers' comments:

Reviewer's Responses to Questions

**Comments to the Author**

Reviewer #1: All comments have been addressed

Reviewer #2: All comments have been addressed

Reviewer #3: (No Response)

**********

2. Is the manuscript technically sound, and do the data support the conclusions?

Reviewer #1: Yes

Reviewer #2: Yes

Reviewer #3: (No Response)

**********

3. Has the statistical analysis been performed appropriately and rigorously?

Reviewer #1: Yes

Reviewer #2: Yes

Reviewer #3: (No Response)

**********

4. Have the authors made all data underlying the findings in their manuscript fully available?

The PLOS Data policy

Reviewer #1: Yes

Reviewer #2: Yes

Reviewer #3: (No Response)

**********

5. Is the manuscript presented in an intelligible fashion and written in standard English?

Reviewer #1: Yes

Reviewer #2: Yes

Reviewer #3: (No Response)

**********

Reviewer #1: The authors have adequately addressed all major concerns raised in my previous review. The revised manuscript is substantially improved in its methodological description, statistical analysis, reporting of limitations, and interpretation of the diagnostic findings. The study is now appropriately presented as an exploratory case-control investigation, and the limitations related to spectrum bias, normalization strategy, repeated control comparisons, internal validation, and lack of external validation are clearly acknowledged.

Reviewer #2: The Authors addressed all the comments satisfactorily.

Although this method has limited clinical relevance due to a significant time lag, it will help in further research.

Reviewer #3: (No Response)

**********

what does this mean?). If published, this will include your full peer review and any attached files.

If you choose “no”, your identity will remain anonymous but your review may still be made public.

**Do you want your identity to be public for this peer review?**  For information about this choice, including consent withdrawal, please see our Privacy Policy

Reviewer #1: **Yes:**  Ebru DERİCİ EKER

Reviewer #2: **Yes:**  Anand Kumar, MD, DM, Associate Professor, Institute Of Medical Sciences, Banaras Hindu University, Varanasi, India

Reviewer #3: **Yes:**  Rameshwar Nath Chaurasia

**********

# Editor report: Acceptance letter

**DOI:** 10.1371/journal.pone.0359648.r004

**Authors:** Francesco Sessa

PONE-D-26-35421R1

PLOS One

Dear Dr. Koshelyuk,

I'm pleased to inform you that your manuscript has been deemed suitable for publication in PLOS One. Congratulations! Your manuscript is now being handed over to our production team.

At this stage, our production department will prepare your paper for publication. This includes ensuring the following:

* All references, tables, and figures are properly cited

* All relevant supporting information is included in the manuscript submission,

* There are no issues that prevent the paper from being properly typeset

You will receive further instructions from the production team, including instructions on how to review your proof when it is ready. Please keep in mind that we are working through a large volume of accepted articles, so please give us a few days to review your paper and let you know the next and final steps.

Lastly, if your institution or institutions have a press office, please let them know about your upcoming paper now to help maximize its impact. If they'll be preparing press materials, please inform our press team within the next 48 hours. Your manuscript will remain under strict press embargo until 2 pm Eastern Time on the date of publication. For more information, please contact onepress@plos.org.

You will receive an invoice from PLOS for your publication fee after your manuscript has reached the completed accept phase. If you receive an email requesting payment before acceptance or for any other service, this may be a phishing scheme. Learn how to identify phishing emails and protect your accounts at https://explore.plos.org/phishing.

If we can help with anything else, please email us at customercare@plos.org.

Thank you for submitting your work to PLOS One and supporting open access.

Kind regards,

PLOS One Editorial Office Staff

on behalf of

Lecturer Francesco Sessa

Academic Editor

PLOS One
